# Network Modeling Reveals Cross Talk of MAP Kinases during Adaptation to Caspofungin Stress in *Aspergillus fumigatus*

**DOI:** 10.1371/journal.pone.0136932

**Published:** 2015-09-10

**Authors:** Robert Altwasser, Clara Baldin, Jakob Weber, Reinhard Guthke, Olaf Kniemeyer, Axel A. Brakhage, Jörg Linde, Vito Valiante

**Affiliations:** 1 Department of Systems Biology/Bioinformatics, Leibniz Institute for Natural Product Research and Infection Biology—Hans Knöll Institute, Adolf-Reichwein-Str. 23, 07745, Jena, Germany; 2 Department of Molecular and Applied Microbiology, Leibniz Institute for Natural Product Research and Infection Biology—Hans Knöll Institute, Adolf-Reichwein-Str. 23, 07745, Jena, Germany; 3 Department of Microbiology and Molecular Biology, Institute of Microbiology, Friedrich Schiller University Jena, Adolf-Reichwein-Str. 23, 07745, Jena, Germany; 4 Integrated Research and Treatment Center, Center for Sepsis Control and Care (CSCC), Jena University Hospital, 07747, Jena, Germany; 5 Leibniz Junior Research Group—Biobricks of Microbial Natural Product Syntheses, Leibniz Institute for Natural Product Research and Infection Biology—Hans Knöll Institute, Adolf-Reichwein-Str. 23, 07745, Jena, Germany; Universidade de Sao Paulo, BRAZIL

## Abstract

Mitogen activated protein kinases (MAPKs) are highly conserved in eukaryotic organisms. In pathogenic fungi, their activities were assigned to different physiological functions including drug adaptation and resistance. *Aspergillus fumigatus* is a human pathogenic fungus, which causes life-threatening invasive infections. Therapeutic options against invasive mycoses are still limited. One of the clinically used drugs is caspofungin, which specifically targets the fungal cell wall biosynthesis. A systems biology approach, based on comprehensive transcriptome data sets and mathematical modeling, was employed to infer a regulatory network and identify key interactions during adaptation to caspofungin stress in *A*. *fumigatus*. Mathematical modeling and experimental validations confirmed an intimate cross talk occurring between the cell wall-integrity and the high osmolarity-glycerol signaling pathways. Specifically, increased concentrations of caspofungin promoted activation of these signalings. Moreover, caspofungin affected the intracellular transport, which caused an additional osmotic stress that is independent of glucan inhibition. High concentrations of caspofungin reduced this osmotic stress, and thus decreased its toxic activity. Our results demonstrated that MAPK signaling pathways play a key role during caspofungin adaptation and are contributing to the paradoxical effect exerted by this drug.

## Introduction

Human fungal pathogens can cross epithelial barriers and grow inside hosts. Consequently, they need to adapt quickly to changes in the environment. Once the cell has sensed environmental changes, signaling cascades activate transcriptional regulators, which modulate the expression of specific target genes. Among the different signaling pathways, the mitogen activated protein kinase (MAPK) cascades have been well studied in fungi [1, 2]. They consist of a conserved module of three kinases, which in turn phosphorylate each other. The bottleneck for each cascade is the single MAPK, which normally moves into the nucleus after being phosphorylated. The major physiological activities assigned to MAPKs in fungi are cell wall biosynthesis, osmoregulation and mating [1, 2]. Additionally, their action was also associated with invasion and pathogenesis in both plant and human pathogenic fungi [1, 2].

*Aspergillus fumigatus* is the most important saprophytic human fungal pathogen. Conidia of this fungus are ubiquitous, and constantly inhaled by humans. In healthy individuals, conidia are quickly removed by the immune system, but in immunocompromised individuals, they can cause systemic infections, including invasive aspergillosis [3, 4].

The *A*. *fumigatus* genome codes for four putative MAPKs [5]. MpkA is the central regulator of the cell wall integrity (CWI) pathway, and its activity was associated with the response to cell wall disturbing compounds and reactive oxygen species [6, 7]. MpkB shares similarities with kinases reported to be involved in mating, but its function has not been elucidated so far. MpkC and the fourth MAPK SakA share similarity with Hog1 from *Saccharomyces cerevisiae* [8], which is the main regulator of the high osmolarity glycerol response (HOG) pathway [9]. SakA was also described to play a role in adaptation to stress caused by the antifungal drug caspofungin [10], while the function of MpkC was more related to signaling required for carbon source utilization than to osmostress response [11].

While the knowledge about the function of MAPKs has increased continuously, there are still many open questions about the cross talk between the different signaling cascades [12]. For this purpose, systems biology can offer an unbiased bird’s eye approach, which can help to detect important cross talks active in cells during the response to external stimuli. In particular, computer simulations integrating current knowledge can be used for large-scale gene, protein and metabolite data sets. Such 'omics' data sets can be processed applying network inference approaches, which are reverse engineering tools used to predict gene interactions [13, 14].

**Fig 1:**
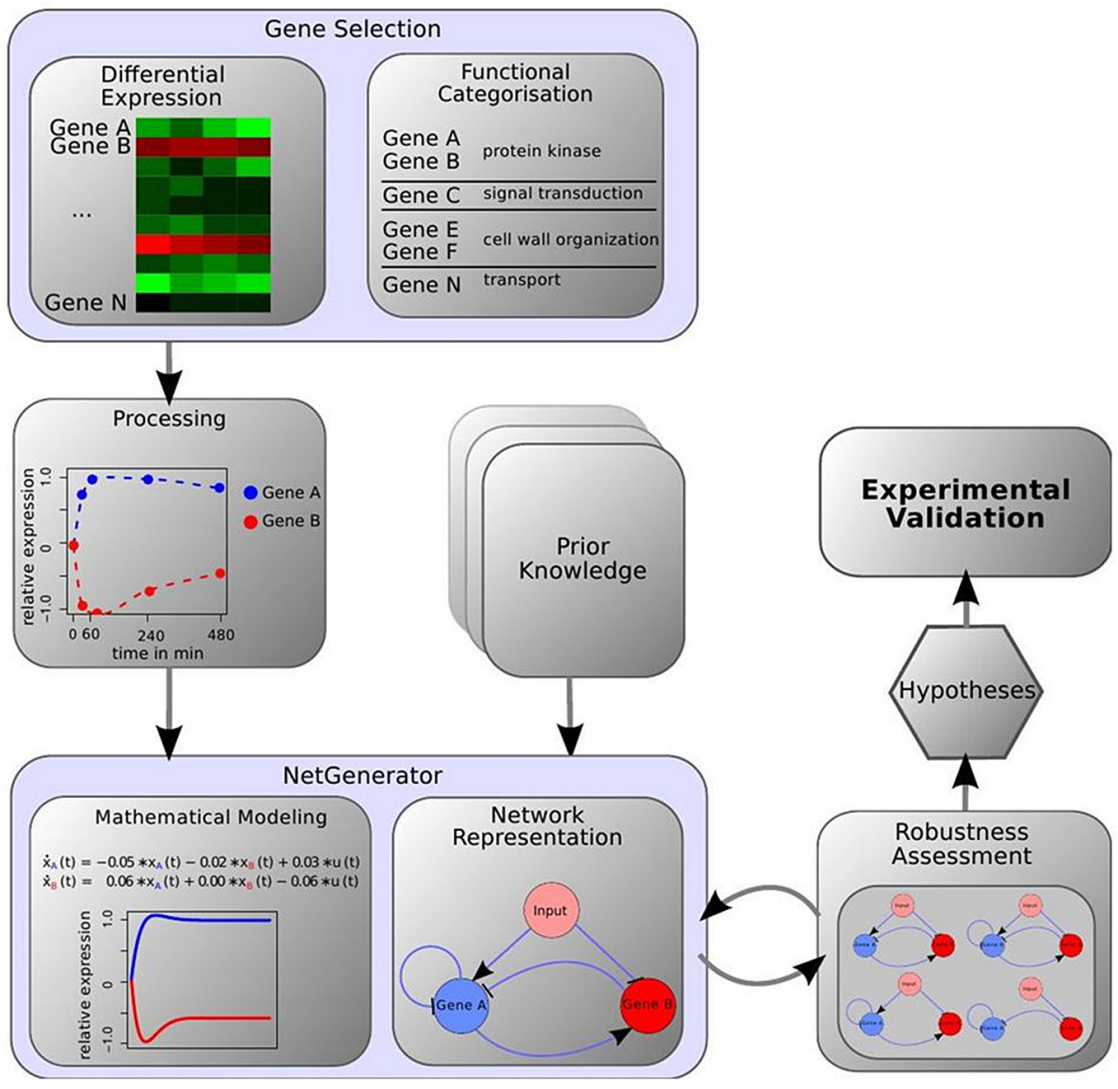
Depiction of the workflow. Genes were selected based on their expression steady-state levels and their assigned function. RNA-Seq data and prior-knowledge were used as inputs for the Net*Gene*rator. Using a mathematical modeling, a network was predicted, which was then evaluated and tested for robustness. A final model was selected, which led to new hypotheses that were experimentally validated.

Net*Gene*rator is a network inference-modeling tool previously applied to infer gene regulatory networks for fungi [15, 16], infected host [16, 17], and both the pathogen and the host during their interaction [18]. The tool uses differential equations to model the temporal change in gene expression (Fig 1) [17]. Additionally, Net*Gene*rator applies the sparseness criterion to only predict those interactions that are indispensable to fit the measured data. Furthermore, it allows integration of prior-knowledge, *i*.*e*., known or hypothetical interactions from additional sources such as literature [19, 20].

For this study, Net*Gene*rator was used to process large RNA-Seq data sets obtained by genome-wide transcriptomics aimed to investigate the response of *A*. *fumigatus* to the stress caused by caspofungin. Caspofungin was the first clinically applied echinocandin (CANCIDAS, caspofungin acetate), which specifically targets the fungal cell wall [21]. In particular, it inhibits the activity of the highly conserved membrane protein Fks1, which is responsible for the synthesis of the major structural compound of the fungal cell wall, the polysaccharide β-(1,3)-glucan [22]. The two main drawbacks of the use of this drug are the emergence of resistant strains, and the occurrence of the so-called paradoxical effect, which describes the phenomenon of reduced activity against fungi at high drug concentrations [23, 24].

RNA-seq analysis revealed that more than 40% of the *A*. *fumigatus* genes were differentially regulated during caspofungin stress. The predicted regulatory network model discovered direct and dynamic interactions between the MAPKs MpkA and SakA. Computational analyses, coupled with experimental proof, revealed that the cross talk between MpkA and SakA plays a major role during adaptation to caspofungin stress. Moreover, caspofungin causes an additional osmotic stress, which is independent of its inhibitory activity on β-(1,3)-glucan biosynthesis, and which is linked to the paradoxical effect exerted by this drug.

## Material and Methods

### Strains and Growth Conditions

*A*. *fumigatus* strains used in this study are listed in S1 Table. For molecular techniques used to obtain mutant strains, please check the supplemental material.

RNA samples for sequence analyses (RNA-seq) were obtained by growing mycelia for 16 h in *Aspergillus* Minimal Medium (AMM), and then addition of caspofungin (0.1 μg ml^-1^). Samples were taken at different time points (0.5 h, 1 h, 4 h and 8 h after treatment). For Δ*sakA* and Δ*mpkA* knock-out mutants samples for RNA-Seq were taken at 1h and 4h after caspofungin treatment. Three biological replicates from each time point were collected for all experiments. As controls, samples were taken before adding caspofungin (0 h).

Mycelia for immunoblots were obtained by growing *A*. *fumigatus* for 16 h in AMM, and then caspofungin was added at the reported concentrations. Samples were taken at different time points (0.5 h, 1 h, 4 h and 8 h after treatment). For the inducible *xyl*p-*fks1* mutants strain, mycelia were obtained after growing for 16 h in AMM with 2% (w/v) xylose, and then mycelia were collected, washed with water. Then, mycelia were cultivated again in fresh media with 1% (w/v) glucose as the carbon source. Samples were collected at different time points mentioned above.

### cDNA Library Construction, Sequencing and Analysis

Total RNA was extracted using the Qiagen RNeasy Plant Mini kit (Qiagen, Germany), according to the manufacturer’s instructions. Total RNA was used for Illumina next-generation sequencing and processed by GATC (Germany). TopHat [25] was used to map the reads to the *A*. *fumigatus* A1163 genome (S1 Text). Structural gene annotation provided by CADRE [26] was utilized to count the number of reads mapped to each gene. The R package DESeq [27, 28] was used to normalize read counts for different gene lengths and library sizes, and to identify differentially expressed genes responding to caspofungin. The expression data for each time point were compared to the control (0 h). Genes were considered differentially expressed when the FDR adjusted p-value was ≤ 0.05 in any of these comparisons. FunCat enrichment analysis was performed using FungiFun [29], also with an adjusted p-value threshold of 0.05. The diagrams were created using R package VennDiagram [30].

### Network Inference

Prior-knowledge was implemented with RNA-Seq data obtained by incubating the Δ*sakA* mutant, the Δ*mpkA* mutant and the recipient strain Δ*akuB* mutant with caspofungin. If a gene *x* was differentially expressed (FDR adjusted p-value ≤ 0.05) in a MAPK mutant strain, a potential interaction between *x* and MAPK was considered. When the expression was higher in the knock-out, inhibition was assumed; otherwise it was classified as activation. The Δ*akuB* strain was used to rule out any influence on the caspofungin stress response by the *akuB* deletion. All prior-knowledge interactions can be found in the S2 Table.

The inference analysis was performed using the Net*Gene*rator tool Version 2.1 [31]. The normalized logarithmic expression values were scaled to range between [-1, +1], in order to compare relative gene expression profiles rather than absolute gene expression. The three different replicates for each time point were considered as independent experiments.

From the dynamic time-resolved data, Net*Gene*rator created an interaction network using differential equations. To infer the change in gene expression *x*_*i*_ of gene *I* the expression of all other genes of the current time point was weighted by coefficients *β*, representing the influence on *i*. A negative weight represented an inhibitory effect, while a positive weight represented an activation of *i*. If the weight was zero, no connection was assumed. Additionally, the effect of the external stimulus was weighted as well. This was done for all time points. The weighted sum of the expression for all the other genes plus the external stimulus was used to simulate the gene expression of *i*.

The robustness of the network was further increased by cross-validation of the prior-knowledge and tested *via* adding artificial noise to the measured data. After the inference, the model error was calculated and weighted by the number of coefficients, the network size and the number of implemented prior-knowledge (S1 Text). First comparison was done using the model error and network size, since a model that complies with the sparseness criterion was preferred. After selecting suitable networks of comparable model error, the number of implemented prior-knowledge interaction was compared, in order to have models equivalent to the current knowledge.

### Quantitative Real-Time Reverse-Transcription PCR (qRT-PCR)

qRT-PCR was performed with the StepOnePlus Real-Time PCR System (Applied Biosystems), using myTaq HS Mix 2x (Bioline) and Evagreen (Biotium). To check primer efficiency, a standard curve was generated, considering seven serial dilutions of genomic DNA of *A*. *fumigatus* in three replicates. All the used primer pairs had efficiencies in the range of 100% (±10%) (S3 Table). *Act1* (actin) of *A*. *fumigatus* was chosen as housekeeping gene. cDNA was generated using the RevertAid Premium kit (Fermentas). The amplification program consisted of an initial denaturation phase at 95°C for 10 min, 40 cycles with 15 seconds at 95°C and 1 min at 60°C and a final step at 95°C for another 15 seconds. Each gene was analyzed from three biological replicates in triplicate. All the results were standardized with the values obtained for the housekeeping gene. To calculate the relative fold change of each gene the 2^-ΔΔCt^ method was used.

### Western Blot Analysis

Protein extraction and western blot analysis was carried out as previously described [6] using a phospho-p38 MAPK (Thr180/Tyr182) antibody to detect the SakA phosphorylated form, a phospho-p44/42 MAPK (Erk1/2) (Thr202/Tyr204) antibody to detect the MpkA phosphorylated form (Cell Signaling Technology), and a γ-tubulin antibody as a reference (yN-20) (Santa Cruz biotechnology, Inc.). Every experiment was repeated at least three times, using independent biological replicates. Additional information is reported in S1 Text.

### Measurement of the Transporter-Mediated Efflux of Rhodamine-123

Conidia (10^6^ ml^-1^) were cultivated for 16 h in AMM at 37°C with 200 rpm. A final concentration of 20 μM of R123 (20 mM stock solution in MeOH) was added to the growing mycelia alone or in combination with caspofungin. Samples were collected at different time points by filtration, using Miracloth filtration material (Merck Millipore, Germany), and washed intensively using ultra filtrated water. Additional water was removed by squeezing the mycelial pellets using blotting paper. The mycelium was ground with mortar and pestle using liquid nitrogen, and collected in microcentrifuge tubes. Each sample was weighted and then dissolved in PBS 1x (5 μl / mg). After thorough mixing, samples were centrifuged for 15 min at maximum speed. Supernatants were collected without disturbing cell debris, and transferred to new tubes. For each sample, 100 μl of supernatant were transferred to a 96 well plate, and fluorescence was directly measured using a TECAN microplate reader according to the manufacturer’s instructions, using the following settings: excitation at 480 nm and emission at 520 nm. Three independent biological replicas were used for each sample.

## Results

### Global mRNA Response of A. fumigatus to Caspofungin Treatment

**Fig 2:**
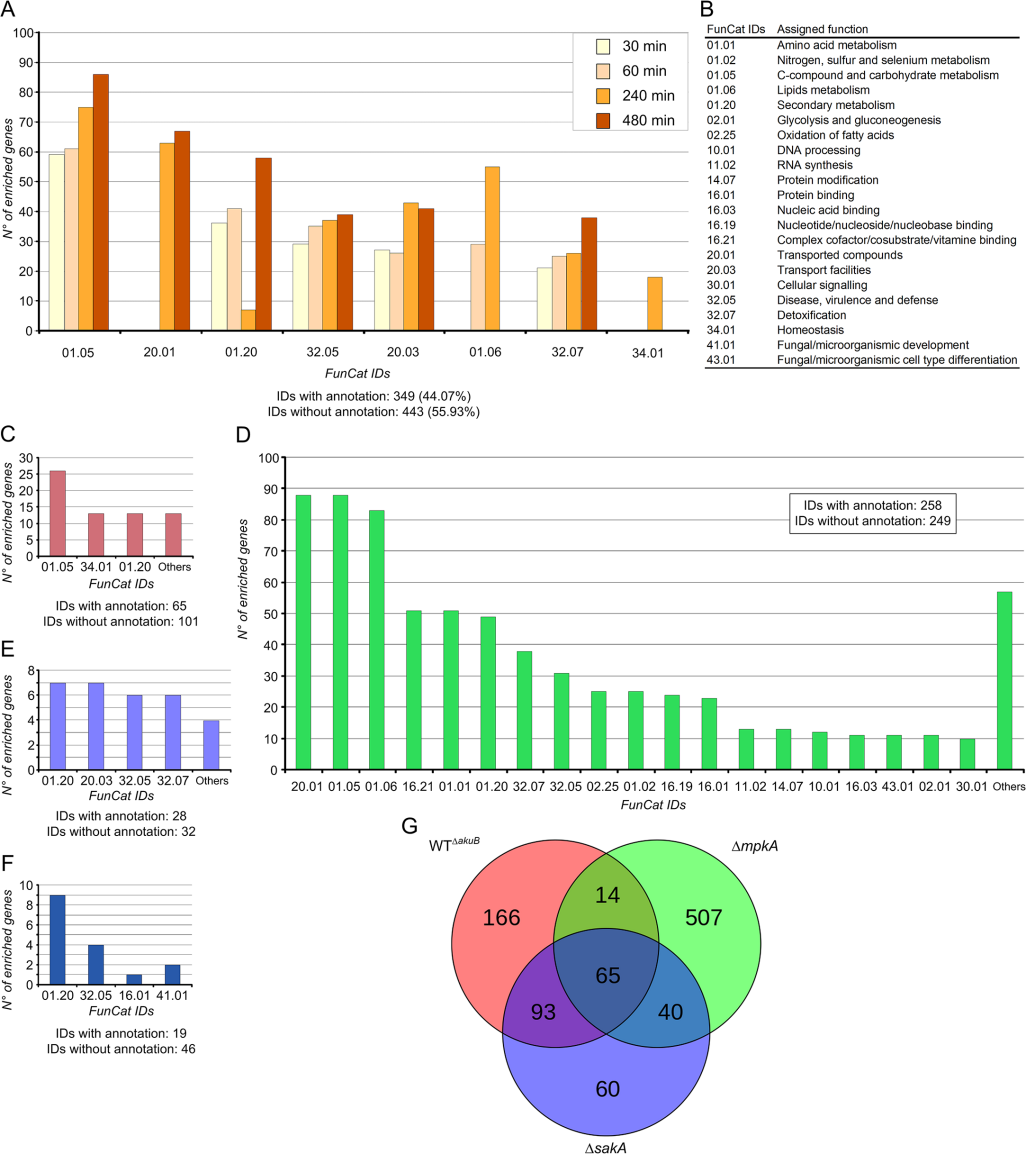
FunCat-enriched categories during caspofungin stress. FunCat-enriched categories were obtained by analyzing the total number of genes differentially expressed in response to caspofungin (0.1 μg ml^-1^). Enrichment analysis using selected genes having log_2_ fold change > 2, and p-value <0.01. The identified FunCat IDs are shown on the x-axis. (A) FunCat-enriched categories identifiedin *A*. *fumigatus* CEA10 wild-type strains during caspofungin stress at the reported time points, and (B) a table showing all the identified categories displayed on the x-axes. Comparative analyses of selected genes in the *A*. *fumigatus* Δ*akuB*, Δ*sakA* and Δ*mpkA* mutant strains are shown. Categorization was implemented by considering differentially regulated genes identified at 3 different time points (0, 1 and 4 h after induction, for all selected mutant strains). In the picture are shown the FunCat-enriched categories uniquely identified in the Δ*akuB* (wt) strain (C), in the Δ*mpkA* strain (D), and in the Δ*sakA* strain (E). Categorization of genes differentially regulated in all strains (F), and the obtained Venn diagram (G), are also shown. Genes used for the analysis are reported in the S2 Database.

*A*. *fumigatus* conidia were pre-cultivated for 16 hours and subsequently stressed by adding caspofungin at subinhibitory concentrations (0.1 μg ml^-1^) [10] directly to the cultivation media. Mycelia were collected after the addition of the drug at the time points 0.5, 1, 4 and 8 h and RNA was extracted subsequently. Approximately 280 million reads were obtained by RNA-Seq, which were mapped to the *A*. *fumigatus* genome, leading to 95% coverage of all annotated genes (S1 Database and S1 Text). Overall, 4,257 differentially expressed genes (DEGs) were identified (FDR adjusted p-value < 0.05), 794 of which showed a fold change log_2_ > 2. Analysis by functional categorization revealed that more than 50% of the identified genes had unknown function, while the remaining ones were scattered and difficult to group in defined categories (Fig 2A and 2B). However, genes putatively involved in carbohydrate metabolism (FunCat ID 01.05) were differentially expressed in response to caspofungin during all time points (*e*.*g*. β-glucosidases, exo- and endo-β-1,3(4)-D-glucanases). This finding confirmed previous results reported for *Aspergillus niger*, in which the exposition to caspofungin induced the cell wall biosynthesis and cell wall reshuffling, thereby mainly affecting sugar metabolism [32]. The study of the dynamic effects of caspofungin at different time points indicated that categories for lipid metabolism (*e*.*g*. C-3 sterol and short chain dehydrogenases) were significantly enriched 1 h and 4 h after induction (ID 01.06), while genes classified as membrane transporters (ID 20.01) were differentially expressed at later time points (4 h and 8 h) (Fig 2A).

### Identification of Candidate Genes and Prior Knowledge for Network Modeling

In order to uncover mechanisms underlying the caspofungin adaptation in *A*. *fumigatus*, the obtained data were processed using the modeling tool Net*Gene*rator. Previous results suggested that Net*Gene*rator is able to predict reliable interactions by using well-defined groups of genes during simulation [16, 18]. Since the number of possible network structures grows exponentially with the number of genes used, a rigorous selection of important genes is necessary. We applied a selection of candidate genes based on technical (differentially expressed) and biological (member of the pathways under study) criteria [16, 18]. According to the modeling capacity, and following the previous functional categorization, we selected 26 differentially expressed genes for modeling that are putatively associated with cell wall biosynthesis, cell membrane stability, glucan synthesis and β-glucosidase activity (Table 1, and S4 Table). Both genes *mpkA* and *sakA* were also considered because of their demonstrated role in caspofungin adaptation [10]. The gene *fks1*, the assigned caspofungin target [33], was also included (Table 1).

**Table 1:** Differentially expressed genes selected for modeling.

			wt / wt_CAS_				Δ*mpkA* / Δ*mpkA*_CAS_		Δ*sakA* / Δ*sakA*_CAS_	
ID	Name	Assigned function	0.5 h	1 h	4 h	8 h	1 h	4 h	1 h	4 h
AFUB_079180	*agnE*_*1*_	mutanase, putative	-2.10	-2.71	-5.80	-5.37	-1.31	-4.25	-2.10	-4.85
AFUB_081470	*agnE*_*2*_	mutanase, putative	-7.73	-4.51	-5.57	-6.19	-0.15	0.55	-1.67	0.57
AFUB_027030	*ags2*	α(1,3)-glucan synthase, putative	-3.81	-2.34	-2.93	-3.12	-1.06	-0.85	-0.50	-0.95
AFUB_053440	*cla4*	serine/threonine kinase activity, putative	-0.15	-0.26	-0.20	-0.33	-0.15	-0.78	0.11	0.05
AFUB_015530	*crf1*	extracellular cell wall glucanase	1.74	2.34	2.30	1.96	-0.39	-1.59	0.72	0.77
AFUB_006160	*exg12*	βglucosidase, putative	-2.52	-1.69	-2.87	-3.92	1.78	1.76	-0.71	-0.62
AFUB_091720	*exg13*	secreted β(1,4)-D-glucan glucan hydrolase	-1.86	-1.83	-2.90	-3.95	-0.27	-1.78	-1.90	-1.65
AFUB_000280	*exg17*	βglucosidase, putative	0.25	1.11	2.21	1.64	0.59	0.67	0.22	0.37
AFUB_078400	*fks1*	β(1,3)-glucan synthase catalytic subunit	-0.00	0.61	0.72	0.07	0.51	0.07	0.42	0.49
AFUB_028470	*gel3*	β(1,3)-glucanosyltransferase	0.03	-0.43	-1.07	-0.73	-1.35	-2.70	-1.13	-2.10
AFUB_081190	*hnm1*	amino acid permease, putative	3.58	2.92	2.03	1.85	1.65	-0.98	2.04	1.11
AFUB_082870	*mae1*	C4-dicarboxylate transporter/malic acid transport protein	-1.11	-2.20	-3.75	-2.02	-0.05	-1.34	-2.05	-2.73
AFUB_012160	*mdr4*	ABC multidrug transporter	-1.75	-2.03	-0.50	-0.48	-0.86	-0.16	-2.19	-1.15
AFUB_022760	*mirC*	siderochrome-iron transporter	-1.12	-0.37	-0.57	-0.44	-2.08	-1.63	-1.89	-1.92
AFUB_070630	*mpkA*	MAP kinase	0.56	0.07	0.33	0.40	-1.23	-0.31	0.34	0.17
AFUB_101270	*ptcH*	protein phosphatase 2C, putative	0.44	1.57	2.52	2.56	3.12	3.35	2.49	3.54
AFUB_020560	*rck2*	calcium/calmodulin-dependent protein kinase, putative	-2.02	-0.61	-1.30	-1.96	0.39	1.46	-0.12	-0.17
AFUB_072830	*rho1*	Rho GTPase	-0.06	0.63	0.43	-0.11	0.22	-0.10	0.24	0.12
AFUB_040580	*rlmA*	SRF-type transcription factor	0.41	0.53	0.50	0.19	0.25	-0.01	-0.05	0.11
AFUB_057130	*rodA*	conidial hydrophobin	-2.81	-0.22	-2.44	-5.24	1.09	2.06	-0.33	0.03
AFUB_016640	*rodB*	conidial hydrophobin	-4.21	-2.79	-5.31	-7.00	-0.80	0.60	-3.97	-3.42
AFUB_012420	*sakA*	MAP kinase	-0.98	-0.58	-0.76	-1.20	0.83	2.13	-1.21	-0.73
AFUB_044820	*sitT*	ABC multidrug transporter	-2.76	-2.99	-1.98	-1.21	-3.04	-3.23	-3.89	-4.23
AFUB_055940	*ssk1*	response regulator, putative	-0.45	0.00	-0.41	-0.78	-1.33	-1.63	-1.12	-0.97
AFUB_010360	*ssk2*	MAP kinase kinase kinase, putative	-0.85	-0.49	-0.10	-0.45	0.09	-0.19	0.38	0.25
AFUB_067390	*ypd1*	phosphotransmitter protein, putative	-0.47	-0.40	-0.33	-0.69	0.02	-0.15	0.03	0.26

The table lists the genes selected for modeling, including accession numbers, and assigned functions. The table also presents the log_2_ fold change values observed for each transcript in different transcriptomics datasets, obtained during caspofungin stress. The log_2_ fold change values were extracted from S1 Database and are referred to the change of the expression patterns after caspofungin (CAS) induction compared to non-induced conditions. Time points after induction are reported in hours (h).

The small number of sufficient species-specific literature made it necessary to generate additional mRNA-Seq data sets that could substitute the missing prior-knowledge. Therefore, the parental strain Δ*akuB*, which was obtained by deletion of a gene coding for a DNA helicase [34], and two knock-out mutant strains lacking either the MAPK gene *sakA* (Δ*sakA*) or *mpkA* (Δ*mpkA*) were exposed to caspofungin (S1 Database, Fugure A, B and C in S1 Fig and S1 Table).

Correlation analysis showed that during caspofungin stress, as expected, the Δ*akuB* strain and the wild-type strain had similar expression profiles. The deletion of the *akuB* gene did not affect the global caspofungin response (Pearson’s correlation coefficient r = 0.97–0.99, Fig 2C and S2 Fig). Comparison of differentially expressed genes identified in the mutant strains after caspofungin induction, revealed that the Δ*mpkA* mutant dramatically changed its global transcriptome during the stress response to caspofungin in comparison to the recipient strain (Fig 2D). Interestingly, these changes did not affect cell wall biosynthesis genes (*e*.*g*. genes involved in chitin or glucan biosynthesis), but were generally related to primary metabolism (*e*.*g*. sugar metabolism) and secondary metabolism (*e*.*g*. genes related to fumicycline biosynthesis) [35]. Additionally, different genes involved in transport (*e*.*g*. ABC and MFS transporters) were down-regulated in the Δ*mpkA* mutant during caspofungin stress. Overall, the caspofungin stress response seems to be enhanced when *mpkA* was lacking. The Δ*sakA* mutant was less reactive to the drug compared to the wild type; in particular no genes related to sugar metabolism were significantly enriched (Fig 2E). In all analyzed strains genes with unknown function were over-represented in the set of differentially regulated genes. It is interesting to note that genes belonging to the biosynthesis gene cluster of the secondary metabolite pseurotin A [36] (grouped in the ID 01.20) were specifically induced by caspofungin (Fig 2F and 2G).

### Prediction of Cross Talk between *sakA* and *mpkA*

**Fig 3:**
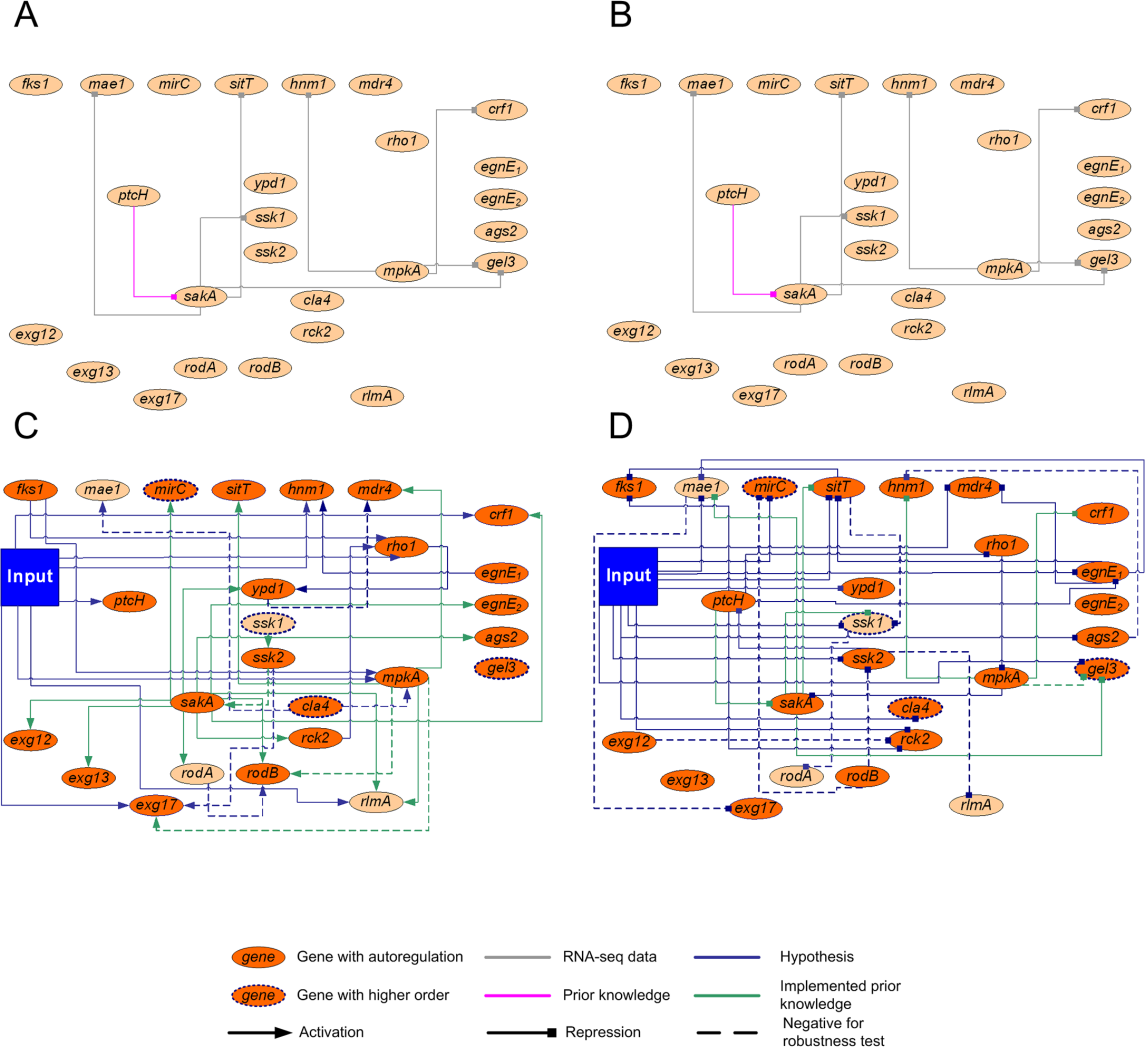
Net*Gene*rator used for modeling prediction. (A) Collected prior-knowledge interactions with activating effects. (B) Collected prior-knowledge interactions with inhibiting effects. (C) Activating and (D) inhibiting interactions of the final predicted network. The legend is shown below the figure. Input marks caspofungin induction. Information about the selected genes is reported in Table 1.

Additional information derived from the Δ*sakA* and Δ*mpkA* mutant strain data sets was used as prior knowledge for modeling. This information led to the discovery of 29 putative interactions, which when added to the 7 literature-based prior-knowledge interactions, resulted in a total of 35 prior-knowledge interactions (Fig 3A and 3B, and S2 Table).

After applying Net*Gene*rator to the selected 26 genes (Table 1) 95 interactions were calculated, including 22 auto-regulatory ones (Fig 3C and 3D). The reliability of the model was evaluated by comparing the kinetics obtained from the simulated gene expression with the transcriptomics-measured expression patterns (S3 Fig). Furthermore, the hypothetical interactions were tested including artificial noise during data processing (S1 Text), with the aim of reducing false positive / negative results. Finally, in order to control that predicted interactions did not only depend on their prior knowledge, a robustness test was performed by randomly skipping parts of prior knowledge. In total, 18 interactions were found either not to be sufficiently robust against noise or heavily depending on prior-knowledge (dotted lines in Fig 3C and 3D).

Among the genes selected for modeling, 7 were predicted as activated by caspofungin stress (Fig 3C), while 11 as repressed (Fig 3D). The two selected MAPKs were predicted as central nodes for caspofungin response, as already indicated in the prior-knowledge analysis. The kinase gene *mpkA* was connected to 6 genes in the network and *sakA* to 15 genes, confirming the biological importance of the MAPK genes (Fig 3C and 3D). The *fks1* gene was not directly affected by the input. On the other hand, a caspofungin-dependent *fks1* repression was predicted *via ptcH* (putative protein phosphatase 2C) [37]. In agreement with the model, *mpkA* was activated by caspofungin and by *fks1*, while *sakA* was repressed *via mpkA* and *ptcH* (Fig 3C). Net*Gene*rator calculation indicated that selected genes coding for transporters (*mae1*, *mdr4*, *sitT* and *mirC*) were negatively affected by caspofungin.

### Validation of Cross Talk between *sakA* and *mpkA*

**Fig 4:**
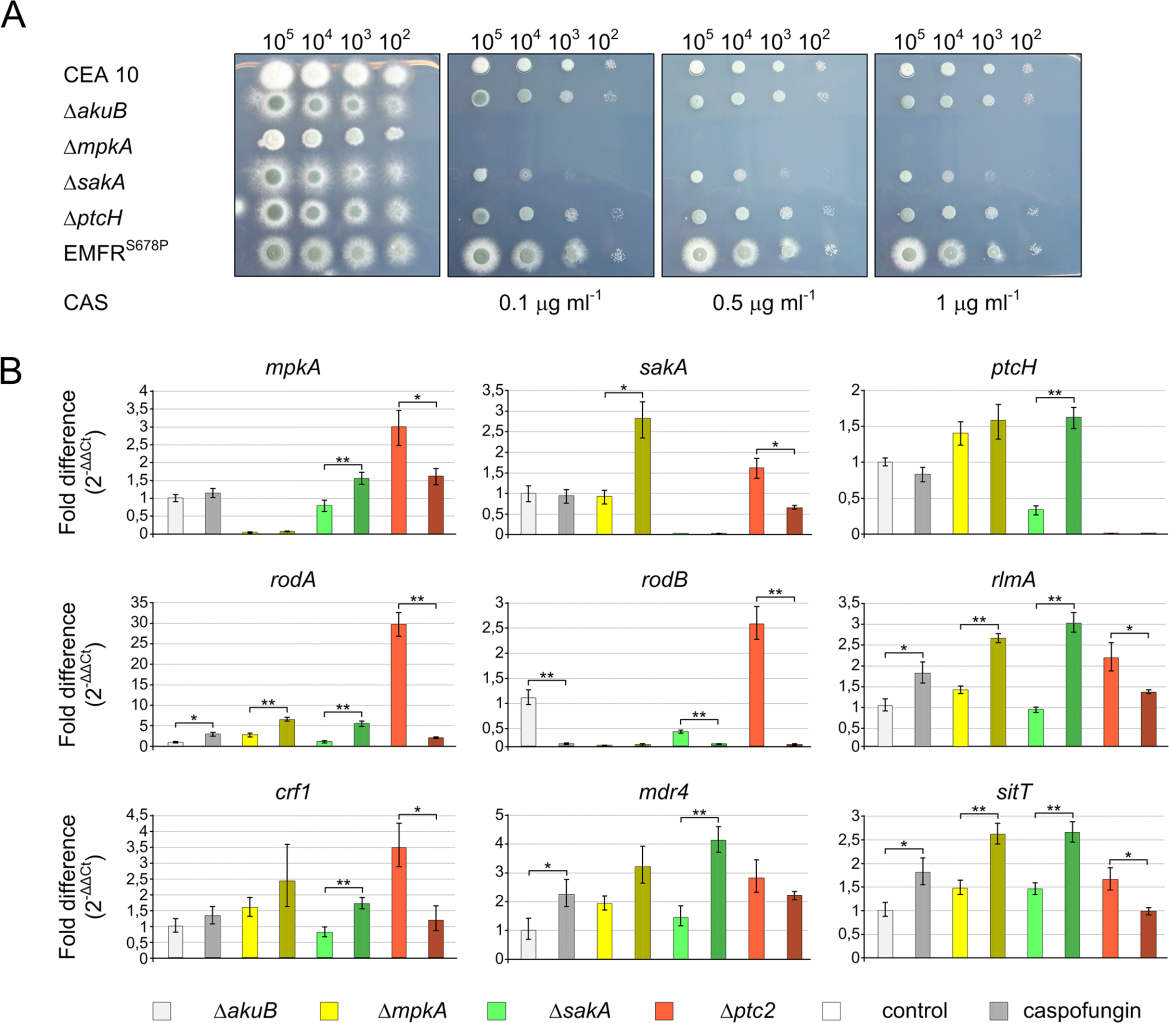
Validation of cross talk between MAPKs. A) The reported strains were tested against caspofungin (CAS) at different concentrations. The indicated number of conidia was spotted on AMM agar plates and incubated at 37°C for 48 hours. B) Results of the qRT-PCR analysis for selected genes in the Δ*akuB*, Δ*mpkA*, Δ*sakA* and Δ*ptcH* mutant strains are shown. Total RNA was extracted before (T0) and 4 h after caspofungin induction (T4). In all performed experiments, the fold changes for each gene were obtained applying the 2^-ΔΔCt^ method (reported in the *y* axes). Data ± SDs. Statistical significance was determined for all the experiments by a Student’s t test. Significance of differences of data with P<0.005 (*) and P<0.001 (**) is indicated. qRT-PCR data for each strain are marked in different colours, as indicated below. Empties bars indicate expression levels for untreated samples, while treated samples are speckled.

To validate the model experimentally, selected genes were re-analyzed by quantitative real-time PCR (qRT-PCR) at two different time points (before and 4 hours after caspofungin induction) on selected mutant strains (Fig 4). *ptcH* was the only putative phosphatase gene differentially regulated during caspofungin stress and predicted by the model that its protein acts as inhibitor of SakA. Because of the importance of phosphatases in regulation of signaling cascades, a Δ*ptcH* mutant strain was generated (Fig D, E and F in S1 Fig). To determine caspofungin effects on the selected strains, different spore concentrations of the wild type and mutants were inoculated on agar plates supplemented with increasing concentrations of caspofungin (Fig 4A). The Δ*mpkA* strain did not grow in the presence of caspofungin, while the Δ*sakA* strain was only inhibited by caspofungin concentrations >0.1 μg ml^-1^, confirming results previously reported [10]. On the other hand, under the tested conditions the Δ*ptcH* strain did not exhibit any different phenotype in comparison to the recipient strain (Fig 4A).

qRT-PCR revealed that in the Δ*mpkA* strain the mRNA steady-state level of *sakA* was increased compared to the wild type (Fig 4B). This suggests a repression of *sakA* by *mpkA*, as predicted in the computational model (Fig 3D). We also analyzed genes connected to caspofungin stress and to both MAPKs (Fig 3C and 3D). These genes code for the glucan synthase Crf1, the putative ABC multidrug transporters Mdr4 and SitT, and the transcription factor RlmA, which was reported to be involved in the maintenance of cell wall integrity in the close related species *Aspergillus nidulans* [38]. mRNA steady-state levels of these genes were induced by caspofungin (Fig 4B).

As shown in Fig 4B, in the Δ*ptcH* mutant strain the expression of all analyzed genes was up-regulated in the absence of caspofungin. When caspofungin was added, the target genes showed decreased expression levels. With the only exception of *rodB*, this expression pattern was opposite to the one observed for the Δ*sakA* deletion mutant strain.

The qRT-PCR analysis confirmed the interaction between *mpkA* and *sakA*. However, not all of the analyzed transcripts confirmed the expression levels determined by RNA-seq. These inconsistencies could be explained by the technical differences between the two techniques, as previously reported [39].

Because kinases are mainly regulated at the post-translational level, we studied the phosphorylation of both MpkA and SakA. Western blot analyses were performed using commercially available anti-MpkA and anti-SakA antibodies which specifically detect the phosphorylated form of these kinases. By contrast, the commercially available antibody that should be used to determine the total amount of these kinases, including their unphosphorylated forms, was not specific. Thus, the commercially available anti-γ-tubulin antibody was used to determine the level of this housekeeping protein as a loading control (S5 Table).

**Fig 5:**
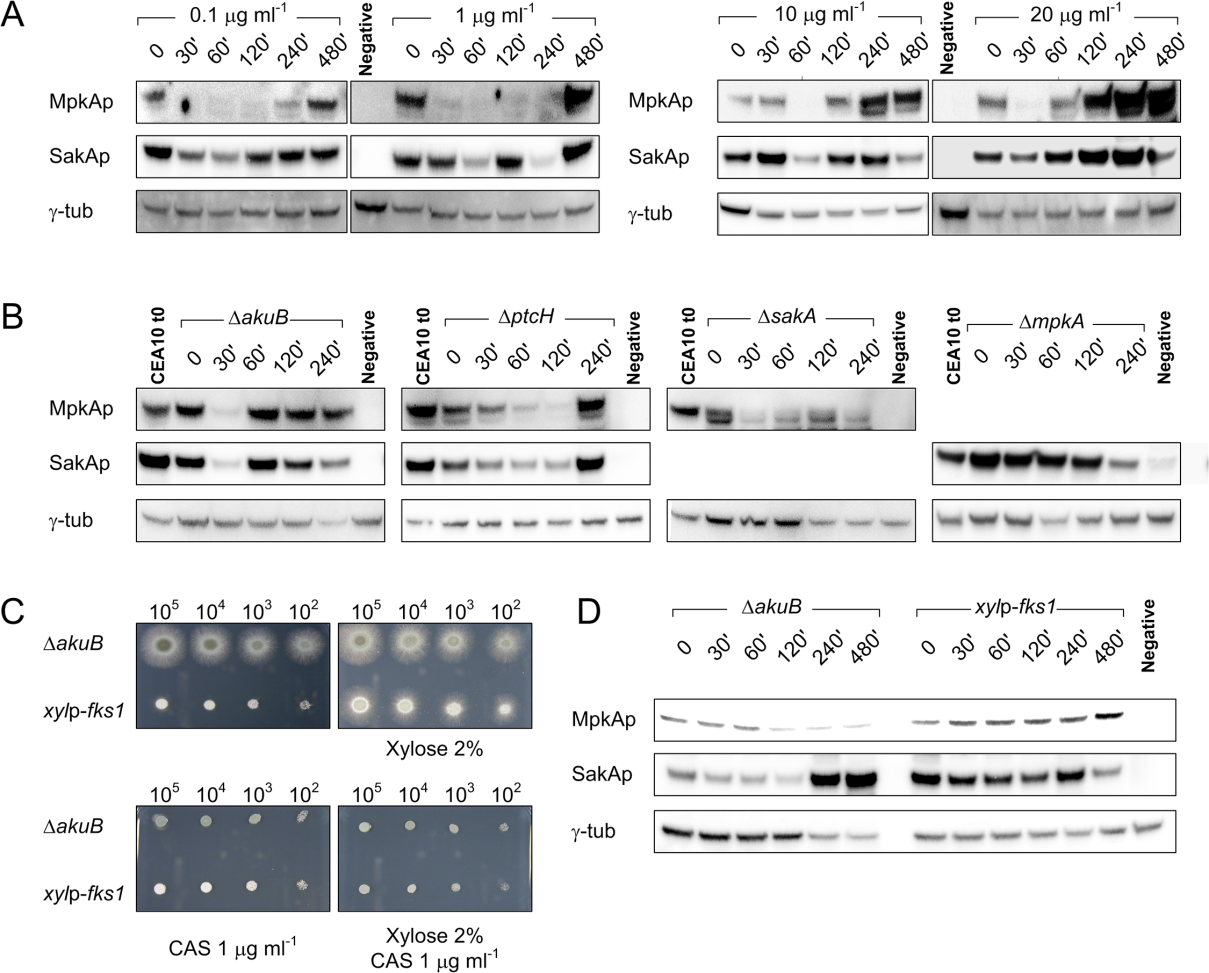
Western blot analysis to determine phosphorylation status of MpkA and SakA during caspofungin stress. (A) Western blot analysis to determine phosphorylation level of MpkA (using an Anti phospho-p42-44 antibody) and SakA (using an Anti phospho-p38 antibody) in *A*. *fumigatus* wild-type strain (using different caspofungin concentrations), and (B) in the Δ*akuB*, Δ*mpkA*, Δ*sakA* and Δ*ptcH* mutant strains, exposed to 0.1 μg ml^-1^ of caspofungin. The γ-tubulin antibody was used as standard loading control in all experiments. Samples were collected at the reported time points (minutes after induction). (C) The recipient strain Δ*akuB* and the inducible *xyl*p-*fks1* mutant strain were tested against caspofungin (CAS) with and without 2% (w/v) xylose in the media. (D) The same strains were grown in liquid media with 2% (w/v) xylose and then transferred in fresh media with 1% (w/v) glucose as the sole carbon source. This experiment was performed to measure the effect of *fks1* repression on MpkA and SakA phosphorylation.

The *A*. *fumigatus* wild-type strain was challenged with different caspofungin concentrations (Fig 5A). Immunoblot analysis showed that the level of phosphorylated MpkA decreased during the period of caspofungin stress, and increased again during caspofungin adaptation (latest time point after 8 h). An increase of MpkA phosphorylation was seen when more than 10 μg ml^-1^ caspofungin was given to the culture; in this case the increase of phosphorylated MpkA was much stronger than the one observed for lower caspofungin concentrations (Fig 5A). A similar picture emerged for SakA phosphorylation, which strongly depended on the concentrations of caspofungin used. In particular, SakA was highly phosphorylated at higher caspofungin concentrations (20 μg ml^-1^) when paradoxical growth occurs, while the use of lower concentrations of caspofungin resulted in constant phosphorylation levels during the investigated time points (Fig 5A).

To further elucidate the role of MAPKs during caspofungin stress, we determined the phosphorylation status of MpkA and SakA in relevant mutant strains in the presence of sub-inhibitory concentrations of caspofungin, similarly to the transcriptomics experiment (Fig 5B). The phosphorylation status of MpkA and SakA in the Δ*akuB* strain was dissimilar to that one observed for the wild-type strain. In particular, the MpkA inhibition was less pronounced compared to the wild-type strain. In the Δ*ptcH* strain, the phosphorylation levels of MpkA and SakA were lower in comparison to the recipient strain (Fig 5B). On the basis of these results we conclude that the phosphatase PtcH positively affects phosphorylation of MpkA and SakA.

Concerning the interaction of the two MAPKs, western blot experiments showed that the lack of *sakA* negatively influenced the level of MpkA phosphorylation after caspofungin induction. The phosphorylation status of MpkA was always lower in the *sakA* mutant than in the wild-type strain (Fig 5B). In contrast, the lack of *mpkA* led to a higher SakA phosphorylation level at earlier time points of caspofungin stress (Fig 5B). This finding suggests that the HOG pathway was also activated during cell-wall stress and contributed to MpkA activation during adaptation to caspofungin stress.

With the aim of determining the effects of Fks1 inhibition on MpkA and SakA, an inducible *xyl*p-*fks1* strain was constructed (Fig G, H, I in S1 Fig). The native *fks1* promoter was substituted by a xylose inducible promoter, which is repressed by glucose, and activated by the presence of xylose [40]. The resulting recombinant strain displayed highly reduced growth in absence of xylose, but not increased susceptibility against caspofungin, which confirmed data previously reported [41]. In order to investigate the effects of *fks1* repression on MAPK phosphorylation, the inducible mutant and the corresponding recipient strain were cultivated in the presence of 2% of xylose for 16 hours, and afterward mycelia was collected, washed, and re-inoculated in fresh AMM media with glucose as sole carbon source. As shown in Fig 4D, the *fks1* repression promotes phosphorylation of both MpkA and SakA during the observed time points.

### Effects of Caspofungin on Membrane Permeability

Network modeling, transcriptomics and qRT-PCR data provided evidence that caspofungin affects membrane transporters (Figs 2 and 3). To analyze changes in the membrane permeability during caspofungin stress, we applied rhodamine-123 (R123) as fluorescent tracer [42]. For this purpose, different osmo-stressors were used to determine whether R123 was differentially transported into the cytosol (S4 Fig). This experiment confirmed that compounds like AmphotericinB, NaCl and KCl are able to promote R123 acquisition in the fungal cells, while the use of polyethylene glycol, which is a widely-used cryoprotectant, inhibits the up-take of R123 (S4 Fig).

**Fig 6:**
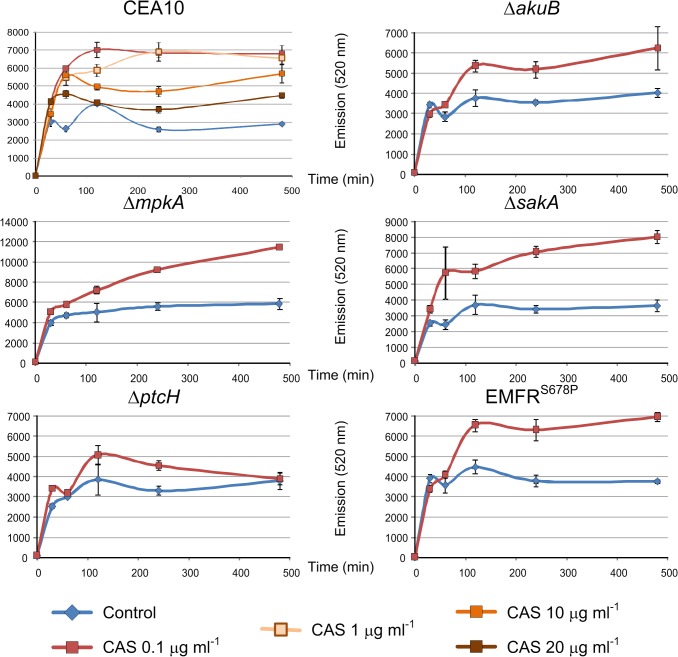
Effects of caspofungin on membrane efflux. Transporter-mediated efflux of rhodamine 123 was determined in absence (blue line) and presence of caspofungin (red line). The wild-type CEA10 strain was challenged with different caspofungin concentrations, while the mutant strains were analyzed using only a caspofungin sub-lethal concentration (0.1 μg ml^-^1). For each sample, cytosolic content was extracted and measured (excitation/ emission 480/520 nm) at the reported time points. ± Standard error of the mean.

*A*. *fumigatus* mycelia were grown for 16 h, and then R123 was added to the media alone or in combination with caspofungin. Measurements of the cytosolic rhodamine content revealed that 4 h after treatment with 0.1 or 1 μg ml^-1^ of caspofungin the fluorescence signal was twice as high in the wild-type strain compared to the caspofungin-untreated control (Fig 6). This indicated that exposure to caspofungin enhanced cell wall permeability. This result is consistent with the differential regulation of genes involved in transport and homeostasis enriched during the FunCat analysis of differentially regulated genes (Fig 2). Unexpectedly, a further increase of caspofungin (≥10 μg ml^-1^) reduced the level of R123 up-take. To elucidate the reason for this phenomenon, which might be linked to the paradoxical effect, we measured the R123 up-take in the mutant strains Δ*mpkA*, Δs*akA* and Δ*ptcH* challenged with of 0.1 μg ml^-1^ of caspofungin. In the Δ*mpkA* mutant, the R123 up-take was higher than in the wild-type strains, both in presence and absence of caspofungin. In the Δs*akA* mutant, the measured fluorescence intensity was slightly higher compared to the wild-type and the recipient strains, but lower than in the Δ*mpkA* strain (Fig 6). In the Δ*ptcH* strain, smaller differences in R123 up-take between caspofungin stress and non-stress conditions were observed. In particular, after 8 h caspofungin induction, there was even no difference detectable between caspofungin-induced and non-induced conditions. Finally, the fluorescence emission measured for the caspofungin resistant mutant EMFR^S678P^, which carries a mutated, caspofungin-resistant β-(1,3)-glucan synthase [24], was similar to that one observed for the Δ*akuB* wild-type strain. This finding suggests that the caspofungin induced osmotic stress was independent from the inhibition of the β-(1,3)-glucan synthase Fks1.

## Discussion

Therapeutic options to cure invasive aspergillosis are still limited and insufficient. Echinocandins are the most recent class of antifungal agents used in the clinics, which specifically target the fungal cell wall. However, since the introduction of the echinocandin caspofungin in clinical trials, no decrease in the mortality rate of patients with invasive aspergillosis has been observed [43]. In contrast to other drugs used to treat mycoses, such as azoles or polyenes, echinocandins have only a fungistatic activity against *A*. *fumigatus* [44]. It was recently demonstrated that this is most likely due to the fact that the deletion of the *fks1* gene, besides giving a strong impaired phenotype, is not lethal for *A*. *fumigatus*. This means that the fungus is also able to survive without β-(1,3)-glucan [41]. Nonetheless, echinocandins are still considered as promising drug candidates for combination therapies because they display neither cytotoxic effects on humans nor *in vitro* antagonism when combined with other antifungal agents [45]. This explains the ongoing activity to test combinatorial therapies of echinocandins with either known or newly isolated active compounds. As an example, a lasso peptide isolated from *Streptomyces humidus* named humidimycin, was recently discovered. It potentiates the antifungal activity of caspofungin, likely by misbalancing MAPKs phosphorylation [10].

In order to better understand the caspofungin stress response in *A*. *fumigatus*, we applied a comprehensive genomic approach based on deep RNA sequencing. The obtained data were analyzed using the state-of-the-art network inference tool Net*Gene*rator [15, 18]. Among the identified and selected genes, Net*Gene*rator assigned central hub-like roles to the MAPK genes *mpkA* and *sakA*. In particular, the calculated model predicted that *mpkA* acted as *sakA* inhibitor during caspofungin stress. Indeed, as shown here, the deletion of *mpkA* had a positive effect on *sakA* expression and phosphorylation.

Previous work in *S*. *cerevisiae* demonstrated that several stress conditions, such as heat shock, oxidative stress, low pH, and zymolase-induced cell wall stress, activated both the HOG and the CWI pathway [46]. Based on findings obtained from the depletion of glucanase activity, it was hypothesized that the CWI signaling pathway acts as a negative regulator of the HOG pathway and that the presence of *hog1* (*sakA* orthologue) is important for the full activation of Slt2 (MpkA orthologous) [47]. These studies already implied that the two pathways are not only functioning in top-down (signal moving from receptor to nucleus), but are connected with each other and are activated from the same stimulus.

Another possibility is that signaling pathways are tuned by the induction or repression of phosphatases, which specifically silence a distinct pathway post-transcriptionally [48]. When analyzing our transcriptomics data, we only identified one gene coding for the putative phosphatase *ptcH*, which was significantly up-regulated during caspofungin stress. It was previously reported that deletion of this gene present a decreased growth during iron starvation stress [37]. However, although in presence of caspofungin a visible phenotype of the Δ*ptcH* mutant was lacking, based on our additional transcription analysis carried out by qRT-PCR we conclude that PtcH formally acts as a repressor of *sakA* and *mpkA* transcription. This is also supported by western blot analysis, which indicated that PtcH did not directly influence the phosphorylation status of SakA and MpkA. It still remains to be clarified how *ptcH* regulates *sakA* and *mpkA* at the transcriptional level.

Immunoblot analysis showed that after caspofungin induction in the Δ*mpkA* mutant a significantly higher phosphorylation level of SakA than in the wild type was detected. At the same time, the MpkA phosphorylation levels dropped during caspofungin treatment in the Δ*sakA* mutant. This finding revealed that the HOG pathway plays a role during caspofungin adaptation, and its activation is important to maintain a fully operational CWI pathway. A functional CWI pathway is necessary to combat and survive caspofungin stress, while both the CWI and the HOG pathways are activated during stress adaptation and are directly connected to the caspofungin paradoxical effect. Indeed, the SakA phosphorylation was strongly induced at caspofungin concentrations higher than 10 μg ml^-1^. Additionally, the activation of these kinases undoubtedly resulted from *fks1* inhibition. The presented data supports the model that a strong Fks1 inhibition either due to high concentrations of caspofungin or to genetic repression of the *fks1* gene, triggers the activation of MpkA and SakA. As previously reported, the β-(1,3)-glucan synthase complex colocalizes with Rho1, which is a small monomeric GTPase that apparently acts in the CWI pathway [49, 50]. This would imply that the CWI is activated by *fks1* repression in a top-down manner.

In parallel, the R123 permeability assay demonstrated that the amounts of caspofungin used were inversely proportional to the intracellular up-take of the molecule. This demonstrates the paradoxical effect exerted by this drug on the level of cellular transport. Juvvadi and coworkers [51] demonstrated that the paradoxical effect exerted by caspofungin in *A*. *fumigatus* is dependent on the activation of calcineurin, which is a calcium-mediated phosphatase. They were able to show that the use of varapaminil, a drug able to block Ca^2+^ channels, erase paradoxical effects, making caspofungin more active. These data fit well with our permeability assay tests. All together, we can conclude that osmobalancing plays a crucial role in caspofungin adaptation.

**Fig 7:**
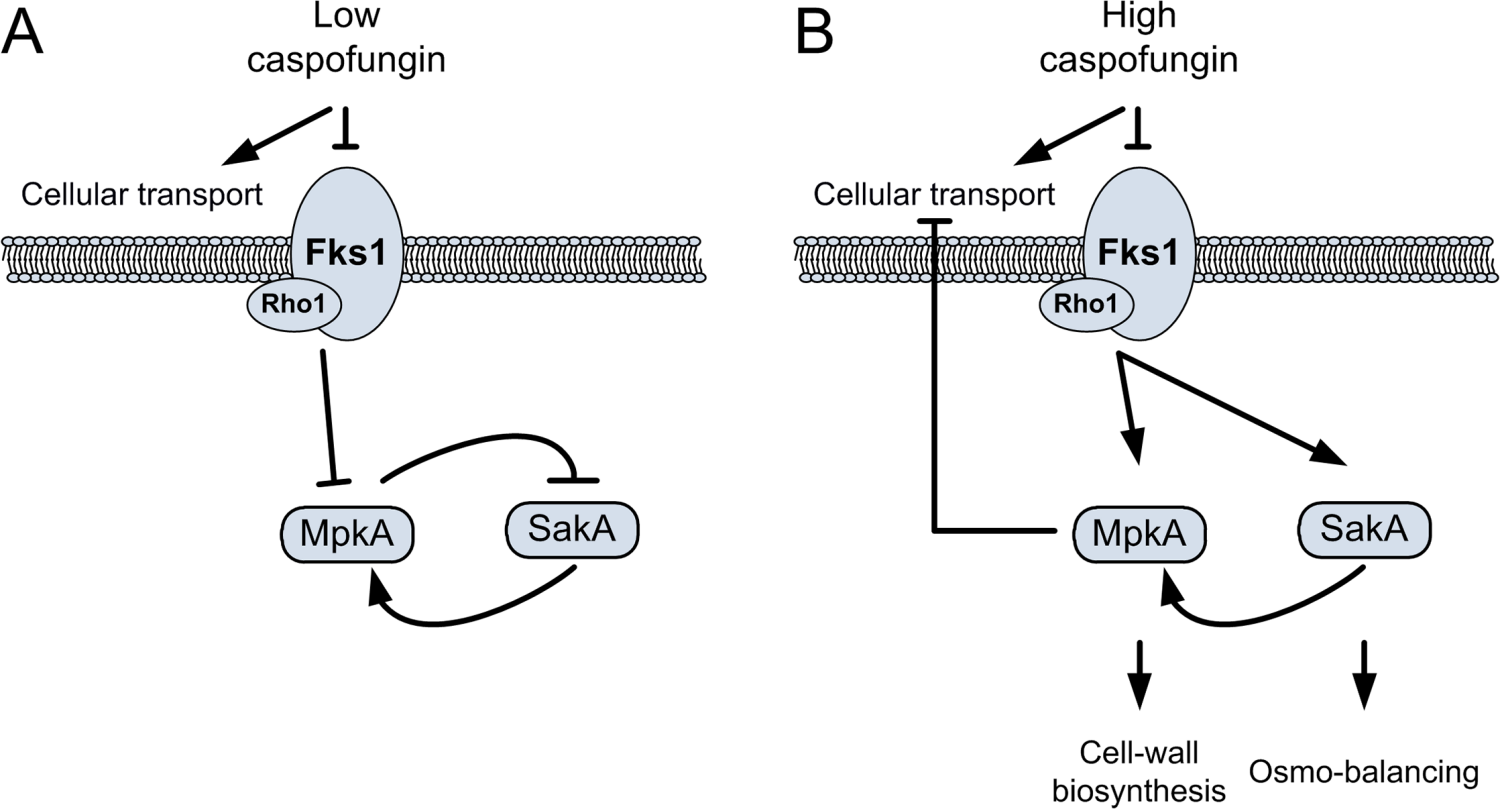
Effects of different caspofungin concentrations on MAPK cascades. (A) When caspofungin is used at inhibitory concentrations (1 μg ml^-1^), a repression of MpkA and SakA phosphorylation levels is observed. Under these conditions, the cellular transport is stimulated and the inhibition of MpkA and SakA avoids the turn-on of compensatory pathways. (B) The use of caspofungin at higher doses strongly activates MpkA and SakA. Under these conditions, cellular transport is inhibited and cell-wall compensatory pathways are more active.

According to our results, we can postulate that the activation of the HOG pathway during caspofungin stress occurs *via* two different ways: as a result of the MpkA repression, and stimulated by the caspofungin-induced osmotic stress (Fig 7A). As a consequence, the use of high doses of caspofungin has two major effects: a decrease of cellular transport, with a likely reduction of caspofungin up-take, and a stronger activation of salvage pathways, such as the CWI and the HOG pathways (Fig 7B).

Taken together, our results demonstrated that the caspofungin response in *A*. *fumigatus* employs two important signaling pathways, the CWI and the HOG pathway. In addition, the CWI pathway is necessary for osmobalancing, which plays a role during caspofungin paradoxical activity. Our data demonstrated that beside the CWI, the HOG pathway represents an additional target that can be used to increase the caspofungin antimycotic activity.

## Supporting Information

S1 Database Analysis of differentially expressed genes in the RNA-Seq databases. (ZIP)

S2 Database Venn diagram groups. **RNA-Seq raw data are available on line at:** http://www.ncbi.nlm.nih.gov/geo/query/acc.cgi?token=qzgdmcmaxranzml&acc=GSE55743.
(XLS)

S1 Fig Generation of *A*. *fumigatus* strains. Generation of deletion plasmids using the yeast transformation-associated recombination (TAR) cloning. A) Schematic representation of the plasmid used for the deletion of the *sakA* locus in the wild-type CEA17 Δ*akuB* recipient strain. B) The entire *sakA* open reading frame was replaced by the *hph* cassette, conferring resistance to hygromycin B. C) Southern blot analysis aimed to confirm *sakA* deletion in the recipient strain. Genomic DNA was digested with *Eco*RV. The probe specifically binds to the *sakA* 3’ flanking region as indicated. D) Schematic representation of the plasmid used for the deletion of *ptcH*. E) The *ptcH* open reading frame was disrupted by the insertion of the *hph* cassette. F) Genomic DNA was digested with *Xho*I. The probe specifically binds to the *ptcH* 5’ flanking region as indicated. G) Schematic representation of the plasmid used for the realization of a *xyl*p-*fks1* inducible strain. H) The inducible *xyl*p promoter was inserted upstream to the *fks1* open reading frame. I) Genomic DNA was digested with *Eco*RV. The probe specifically binds to the *fks1* promoter region, as indicated. Primers are listed in the supplementing S3 Table.
(DOC)

S2 Fig Comparison of log_2_ fold changes for the wild-type (CEA10) and the Δ*akuB* mutant strain. The regression analysis was calculated using differentially expressed genes. A logarithmic read count was used for differentially regulated genes in the wild type (wt) and the Δ*akuB* mutant strain. The correlation between the different expressions for each gene was calculated using the Pearson and Spearman methods in R [27]. The obtained high correlation indicates that the deletion of the *akuB* gene does not have significant effects on global caspofungin response.
(DOC)

S3 Fig Results of the simulation of expression data. In every diagram the x-axis shows the time in minutes and the y-axis the gene expression relative to 0 h scaled to values between [-1, +1]. The dotted lines (red, blue, orange) represent the three replicates for each time point. The solid red line depicts the simulated kinetic.
(DOC)

S4 Fig Using rhodamine 123 (R123) to measure membrane efflux. Transporter-mediated efflux of R123 was determined in absence (blue line) and presence of different osmostress inducers (AmphotericinB [AmpB], NaCl, KCl, Polyethylene glycol [PEG], and caspofungin [CAS]). For each sample, cytosolic content was extracted and measured (excitation/ emission 480/520 nm) at the reported time points. ± Standard error of the mean is reported.
(DOCX)

S1 Table *A*. *fumigatus* strains used in this study. (DOC)

S2 Table List of prior-knowledge used in this work. The table contains the standard names for the regulators, targets, whether the interaction was activating/inhibiting as well as the confidence score that was assigned to it. The column “implemented” shows whether the interaction was found in the final model. The column “source” indicates the resource of the used prior knowledge.
(DOC)

S3 Table Oligonucleotides used in this study. (DOC)

S4 Table List of genes selected for network inference. In the table, systematic names, standard names, description of functions as well as the corresponding GO-categories are listed. The table also indicates whether these genes were previously reported in literature as being part of the response pathway (see reviews from Rispail *et al*. 2009 and Hamel *et al*. 2012)[1, 2]. The FDR adjusted p-values for different comparisons are listed, and are referred to the expression patterns after caspofungin (CAS) induction compared to non-induced conditions. Time points after induction are reported in hours (h).
(DOC)

S5 Table Statistical analysis of signal intensities obtained during immune blots experiments. Signals of kinases phosphorylation and the γ-tubulin (the standard loading control) were quantified by the software Bio-1D (Vilber Lourmat Deutschland GmbH) and the MAP kinase/ γ-tubulin ratio was calculated. Data are presented as the means ± S.E. from three independent experiments. In green are highlighted values where the change of the relative signal was > 1.5, while in orange are reported changes < 0.66. In all experiments, the not induced wild type strain CEA10 was used as control reference, with the exception of the western blots performed to evaluate the effects of the silencing of the *fks1* gene (shift xylose/glucose); in this case the signal detected in the recipient strain Δ*akuB* growth on xylose served as reference.
(DOC)

S1 Text Mapping of transcription data (additional details about the used protocol for analysis of transcriptome data); Network inference (additional details on error calculation and parameter setting; Protocol for western blot analysis. (additional details about the used protocol for immunoblot analysis and image acquisition).
(DOC)
