# Supplementary material for: Network Modeling Reveals Cross Talk of MAP Kinases during Adaptation to Caspofungin Stress in Aspergillus fumigatus
Source: PLoS One. 2015 Sep 10;10(9):e0136932. doi: 10.1371/journal.pone.0136932 (PMC4565559; doi:10.1371/journal.pone.0136932)
Supplement: S1 Fig — Generation of deletion plasmids using the yeast transformation-associated recombination (TAR) cloning. A) Schematic representation of the plasmid used for the deletion of the sakA locus in the wild-type CEA17 ΔakuB recipient strain. B) The entire sakA open reading frame was replaced by the hph cassette, conferring resistance to hygromycin B. C) Southern blot analysis aimed to confirm sakA deletion in the recipient strain. Genomic DNA was digested with EcoRV. The probe specifically binds to the sakA 3’ flanking region as indicated. D) Schematic representation of the plasmid used for the deletion of ptcH. E) The ptcH open reading frame was disrupted by the insertion of the hph cassette. F) Genomic DNA was digested with XhoI. The probe specifically binds to the ptcH 5’ flanking region as indicated. G) Schematic representation of the plasmid used for the realization of a xylp-fks1 inducible strain. H) The inducible xylp promoter was inserted upstream to the fks1 open reading frame. I) Genomic DNA was digested with EcoRV. The probe specifically binds to the fks1 promoter region, as indicated. Primers are listed in the supplementing S3 Table. (DOC) [file pone.0136932.s003.doc]

**S1 Fig. Generation of *A. fumigatus* strains.** Generation of deletion plasmids using the yeast transformation-associated recombination (TAR) cloning. A) Schematic representation of the plasmid used for the deletion of the *sakA* locus in the wild-type CEA17 Δ*akuB* recipient strain. B) The entire *sakA* open reading frame was replaced by the *hph* cassette, conferring resistance to hygromycin B. C) Southern blot analysis aimed to confirm *sakA* deletion in the recipient strain. Genomic DNA was digested with *Eco*RV. The probe speciﬁcally binds to the *sakA* 3’ flanking region as indicated. D) Schematic representation of the plasmid used for the deletion of *ptcH*. E) The *ptcH* open reading frame was disrupted by the insertion of the *hph* cassette. F) Genomic DNA was digested with *Xho*I. The probe speciﬁcally binds to the *ptcH* 5’ flanking region as indicated. G) Schematic representation of the plasmid used for the realization of a *xyl*p-*fks1* inducible strain. H) The inducible *xyl*p promoter was inserted upstream to the *fks1* open reading frame. I) Genomic DNA was digested with *Eco*RV. The probe speciﬁcally binds to the *fks1* promoter region, as indicated. Primers are listed in the supplementing S2 Table.
