# Supplementary material for: Network Modeling Reveals Cross Talk of MAP Kinases during Adaptation to Caspofungin Stress in Aspergillus fumigatus
Source: PLoS One. 2015 Sep 10;10(9):e0136932. doi: 10.1371/journal.pone.0136932 (PMC4565559; doi:10.1371/journal.pone.0136932)
Supplement: S2 Fig — The regression analysis was calculated using differentially expressed genes. A logarithmic read count was used for differentially regulated genes in the wild type (wt) and the ΔakuB mutant strain. The correlation between the different expressions for each gene was calculated using the Pearson and Spearman methods in R [27]. The obtained high correlation indicates that the deletion of the akuB gene does not have significant effects on global caspofungin response. (DOC) [file pone.0136932.s004.doc]

**S2 Fig. Comparison of log_2_ fold changes for the wild-type (CEA10) and the ∆*akuB* mutant strain.** The regression analysis was calculated using differentially expressed genes. A logarithmic read count was used for differentially regulated genes in the wild type (wt) and the Δ*akuB* mutant strain. The correlation between the different expressions for each gene was calculated using the Pearson and Spearman methods in R . The obtained high correlation indicates that the deletion of the *akuB* gene does not have significant effects on global caspofungin response.

1. R Development Core Team. R: A Language and Environment for Statistical Computing. Vienna, Austria : the R Foundation for Statistical Computing. ISBN: 3-900051-07-0. Available online at http://www.R-project.org/.
