# Supplementary material for: Network Modeling Reveals Cross Talk of MAP Kinases during Adaptation to Caspofungin Stress in Aspergillus fumigatus
Source: PLoS One. 2015 Sep 10;10(9):e0136932. doi: 10.1371/journal.pone.0136932 (PMC4565559; doi:10.1371/journal.pone.0136932)
Supplement: S3 Fig — In every diagram the x-axis shows the time in minutes and the y-axis the gene expression relative to 0 h scaled to values between [-1, +1]. The dotted lines (red, blue, orange) represent the three replicates for each time point. The solid red line depicts the simulated kinetic. (DOC) [file pone.0136932.s005.doc]

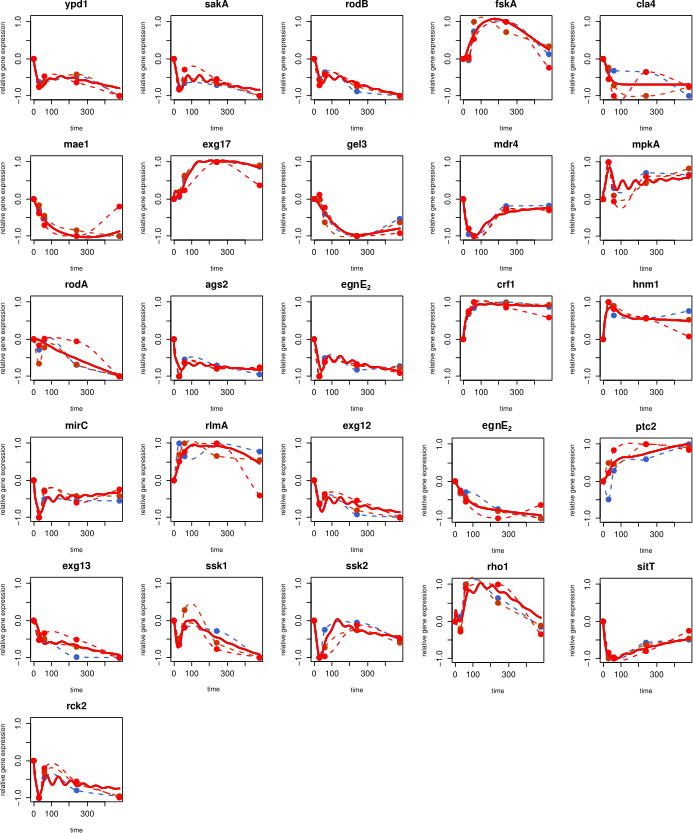


**S3 Fig.** **Results of the simulation of expression data.** In every diagram the x-axis shows the time in minutes and the y-axis the gene expression relative to 0 h scaled to values between [-1, +1]. The dotted lines (red, blue, orange) represent the three replicates for each time point. The solid red line depicts the simulated kinetic.
