# Supplementary material for: Network Modeling Reveals Cross Talk of MAP Kinases during Adaptation to Caspofungin Stress in Aspergillus fumigatus
Source: PLoS One. 2015 Sep 10;10(9):e0136932. doi: 10.1371/journal.pone.0136932 (PMC4565559; doi:10.1371/journal.pone.0136932)
Supplement: S4 Fig — Transporter-mediated efflux of R123 was determined in absence (blue line) and presence of different osmostress inducers (AmphotericinB [AmpB], NaCl, KCl, Polyethylene glycol [PEG], and caspofungin [CAS]). For each sample, cytosolic content was extracted and measured (excitation/ emission 480/520 nm) at the reported time points. ± Standard error of the mean is reported. (DOCX) [file pone.0136932.s006.docx]

**S4 Fig. Using rhodamine 123 (R123) to measure membrane efflux.** Transporter-mediated efflux of R123 was determined in absence (blue line) and presence of different osmostress inducers (AmphotericinB [AmpB], NaCl, KCl, Polyethylene glycol [PEG], and caspofungin [CAS]). For each sample, cytosolic content was extracted and measured (excitation/ emission 480/520 nm) at the reported time points. ± Standard error of the mean is reported.
