# Supplementary material for: Network Modeling Reveals Cross Talk of MAP Kinases during Adaptation to Caspofungin Stress in Aspergillus fumigatus
Source: PLoS One. 2015 Sep 10;10(9):e0136932. doi: 10.1371/journal.pone.0136932 (PMC4565559; doi:10.1371/journal.pone.0136932)
Supplement: S1 Table — (DOC) [file pone.0136932.s007.doc]

**S1 Table. *A. fumigatus* strains used in this study.**

| **Strain** | **Assigned function** | **Genetic background** | Genotype | **Reference** |
| --- | --- | --- | --- | --- |
| CEA10 | Wild type |  |  |  |
| Δ*akuB* | Ku DNA helicase | CEA17 | *akuB*Δ::*pyrG*; PyrG^+^ |  |
| Δ*mpkA* | MAP kinase (MpkA) | CEA17 Δ*akuB* | *akuB*Δ; *mpkA*::*ptrA*; Ptr^+^ |  |
| Δ*sakA* | MAP kinase (Osm1) | CEA17 Δ*akuB* | *akuB*Δ; *sakA*::*hph*; Hyg^R+^ | This study |
| Δ*ptcH* | response regulator | CEA17 Δ*akuB* | *akuB*Δ; *ptc2*::*hph*; Hyg^R+^ | This study |
| EMFR^S678P^ | 1,3-beta-glucan synthase | CEA17 Δ*akuB* | *akuB*Δ; *fks1*::*fks1*(S678F)*/pyrG*; PyrG^+^ |  |

1. da Silva Ferreira ME, Kress MR, Savoldi M, Goldman MH, Hartl A, Heinekamp T, et al. The *akuB*(KU80) mutant deficient for nonhomologous end joining is a powerful tool for analyzing pathogenicity in *Aspergillus fumigatus*. Eukaryot Cell. 2006;5(1):207-11.

2. Valiante V, Jain R, Heinekamp T, Brakhage AA. The MpkA MAP kinase module regulates cell wall integrity signaling and pyomelanin formation in Aspergillus fumigatus. Fungal Genet Biol. 2009;46(12):909-18.

3. Rocha EM, Garcia-Effron G, Park S, Perlin DS. A Ser678Pro substitution in Fks1p confers resistance to echinocandin drugs in *Aspergillus fumigatus*. Antimicrob Agents Chemother. 2007;51(11):4174-6.
