# Supplementary material for: Network Modeling Reveals Cross Talk of MAP Kinases during Adaptation to Caspofungin Stress in Aspergillus fumigatus
Source: PLoS One. 2015 Sep 10;10(9):e0136932. doi: 10.1371/journal.pone.0136932 (PMC4565559; doi:10.1371/journal.pone.0136932)
Supplement: S2 Table — The table contains the standard names for the regulators, targets, whether the interaction was activating/inhibiting as well as the confidence score that was assigned to it. The column “implemented” shows whether the interaction was found in the final model. The column “source” indicates the resource of the used prior knowledge. (DOC) [file pone.0136932.s008.doc]

**S2 Table. List of prior-knowledge used in this work.**

| **Regulator** | **Target** | **Direction** | **Confidence** | **Source** | **Implemented** |
| --- | --- | --- | --- | --- | --- |
| ***mirc*** | *cla4* | activation | 0.66 |  | yes |
| ***mpkA*** | *rlmA* | activation | 0.66 |  | yes |
| ***mpkA*** | *exg12* | activation | 0.5 | Diff. expr.in the ∆mpkA mutant | no |
| ***mpkA*** | *exg17* | activation | 0.5 | Diff. expr.in the ∆mpkA mutant | no |
| ***mpkA*** | *rck2* | activation | 0.5 | Diff. expr.in the ∆mpkA mutant | no |
| ***mpkA*** | *rodB* | activation | 0.5 | Diff. expr.in the ∆mpkA mutant | yes |
| ***mpkA*** | *crf1* | inhibition | 0.5 | Diff. expr.in the ∆mpkA mutant | yes |
| ***mpkA*** | *agnE_2_* | activation | 0.5 | Diff. expr.in the ∆mpkA mutant | no |
| ***mpkA*** | *mirC* | activation | 0.5 | Diff. expr.in the ∆mpkA mutant | no |
| ***mpkA*** | *rlmA* | activation | 0.5 | Diff. expr.in the ∆mpkA mutant | yes |
| ***mpkA*** | *mdr4* | activation | 0.5 | Diff. expr.in the ∆mpkA mutant | yes |
| ***mpkA*** | *sitT* | activation | 0.5 | Diff. expr.in the ∆mpkA mutant | yes |
| ***mpkA*** | *ags2* | activation | 0.5 | Diff. expr.in the ∆mpkA mutant | no |
| ***mpkA*** | *axg13* | activation | 0.5 | Diff. expr.in the ∆mpkA mutant | no |
| ***mpkA*** | *hnm1* | inhibition | 0.5 | Diff. expr.in the ∆mpkA mutant | yes |
| ***mpkA*** | *gel3* | inhibition | 0.5 | Diff. expr.in the ∆mpkA mutant | yes |
| ***ptcH*** | *sakA* | inhibition | 0.66 |  | yes |
| ***sakA*** | *rlmA* | activation | 0.66 |  | yes |
| ***sakA*** | *ssk1* | inhibition | 0.5 | Diff. expr.in the ∆sakA mutant | no |
| ***sakA*** | *gel3* | inhibition | 0.5 | Diff. expr.in the ∆sakA mutant | yes |
| ***sakA*** | *sitT* | inhibition | 0.5 | Diff. expr.in the ∆sakA mutant | no |
| ***sakA*** | *ags2* | activation | 0.5 | Diff. expr.in the ∆sakA mutant | yes |
| ***sakA*** | *exg13* | activation | 0.5 | Diff. expr.in the ∆sakA mutant | yes |
| ***sakA*** | *exg12* | activation | 0.5 | Diff. expr.in the ∆sakA mutant | yes |
| ***sakA*** | *mae1* | inhibition | 0.5 | Diff. expr.in the ∆sakA mutant | yes |
| ***sakA*** | *rck2* | activation | 0.5 | Diff. expr.in the ∆sakA mutant | yes |
| ***sakA*** | *rodA* | activation | 0.5 | Diff. expr.in the ∆sakA mutant | yes |
| ***sakA*** | *rodB* | activation | 0.5 | Diff. expr.in the ∆sakA mutant | yes |
| ***sakA*** | *crf1* | activation | 0.5 | Diff. expr.in the ∆sakA mutant | no |
| ***sakA*** | *agnE_2_* | activation | 0.5 | Diff. expr.in the ∆sakA mutant | yes |
| ***sakA*** | *mirC* | activation | 0.5 | Diff. expr.in the ∆sakA mutant | yes |
| ***sakA*** | *ypd1* | activation | 0.5 | Diff. expr.in the ∆sakA mutant | no |
| ***sakA*** | *cla4* | inhibition | 0.5 | Diff. expr.in the ∆sakA mutant | yes |
| ***ssk1*** | *ssk2* | activation | 0.66 |  | yes |
| ***ssk2*** | *sakA* | activation | 0.66 |  | no |
| ***ypd1*** | *ssk1* | activation | 0.66 |  | no |

The table contains the standard names for the regulators, targets, whether the interaction was activating/inhibiting as well as the confidence score that was assigned to it. The column “implemented” shows whether the interaction was found in the final model. The column “source” indicates the resource of the used prior knowledge.

Table references

1. Haas H, Schoeser M, Lesuisse E, Ernst JF, Parson W, et al. (2003) Characterization of the Aspergillus nidulans transporters for the siderophores enterobactin and triacetylfusarinine C. Biochem J 371: 505-513.

2. Fujioka T, Mizutani O, Furukawa K, Sato N, Yoshimi A, et al. (2007) MpkA-Dependent and -independent cell wall integrity signaling in Aspergillus nidulans. Eukaryot Cell 6: 1497-1510.

3. Dodou E, Treisman R (1997) The Saccharomyces cerevisiae MADS-box transcription factor Rlm1 is a target for the Mpk1 mitogen-activated protein kinase pathway. Mol Cell Biol 17: 1848-1859.

4. Young C, Mapes J, Hanneman J, Al-Zarban S, Ota I (2002) Role of Ptc2 type 2C Ser/Thr phosphatase in yeast high-osmolarity glycerol pathway inactivation. Eukaryot Cell 1: 1032-1040.

5. Hahn JS, Thiele DJ (2002) Regulation of the Saccharomyces cerevisiae Slt2 kinase pathway by the stress-inducible Sdp1 dual specificity phosphatase. J Biol Chem 277: 21278-21284.

6. Posas F, Saito H (1998) Activation of the yeast SSK2 MAP kinase kinase kinase by the SSK1 two-component response regulator. EMBO J 17: 1385-1394.

7. Maeda T, Wurgler-Murphy SM, Saito H (1994) A two-component system that regulates an osmosensing MAP kinase cascade in yeast. Nature 369: 242-245.

8. Posas F, Wurgler-Murphy SM, Maeda T, Witten EA, Thai TC, et al. (1996) Yeast HOG1 MAP kinase cascade is regulated by a multistep phosphorelay mechanism in the SLN1-YPD1-SSK1 "two-component" osmosensor. Cell 86: 865-875.
