# Supplementary material for: Network Modeling Reveals Cross Talk of MAP Kinases during Adaptation to Caspofungin Stress in Aspergillus fumigatus
Source: PLoS One. 2015 Sep 10;10(9):e0136932. doi: 10.1371/journal.pone.0136932 (PMC4565559; doi:10.1371/journal.pone.0136932)
Supplement: S3 Table — (DOC) [file pone.0136932.s009.doc]

**S3 Table. Oligonucleotides used in this study**.

| **Nr.** | **Oligonucleotide** | **Sequence 5´-3´** | **Use** |
| --- | --- | --- | --- |
| **1** | sakA_P1 | **GCTGTAATACGACTCACTATAGGGAATATT**GTCTGTCTAAGGCAATATCG | Cloning |
| **2** | sakA_P2 | **CAATAGTGCCACGTTCTAAATTCAACCAAG**TCTGGGCACGCACGAACTCG | Cloning |
| **3** | sakA_P3 | **CCCAGCACTCGTCCGAGGGCAAAGGAATAG**GAAGGAGGCGTGGCCCAAGC | Cloning |
| **4** | sakA_P4 | **CATAACTAATTACATGATGCGGCCCTCTAG**CTATGGAGTGATCCCCGTCG | Cloning |
| **5** | Hph_For | CTTGGTTGAATTTAGAACGTGG | Cloning |
| **6** | Hph_Rev | CTATTCCTTTGCCCTCGGACGAG | Cloning |
| **7** | ptc2_P1 | **GCTGTAATACGACTCACTATAGGGAATATT**CTTCTGACAGGCACGATTGG | Cloning |
| **8** | ptc2_P2 | **CAATAGTGCCACGTTCTAAATTCAACCAAG**GAGCCACTGACTCTCATTAG | Cloning |
| **9** | ptc2_P3 | **CCCAGCACTCGTCCGAGGGCAAAGGAATAG**CAAAGCGACACATCAGTGAG | Cloning |
| **10** | ptc2_P4 | **CATAACTAATTACATGATGCGGCCCTCTAG**GGCATCAAACTCGTTTACTGTG | Cloning |
| **11** | oJW0083 | **GCTGTAATACGACTCACTATAGGGAATATT**CAACGCCTGCATCATGTTC | Cloning |
| **12** | oJW0084 | **CAATAGTGCCACGTTCTAAATTCAACCAAG**GGGCTGGAATGAAACTGTTGACAG | Cloning |
| **13** | oJW0085 | **ATTTTCATTCATCGACTCGAAGAACCAACC**ATGTCGGGATATCAACAAGGG | Cloning |
| **14** | oJW0086 | **CATAACTAATTACATGATGCGGCCCTCTAG**CAAGCTGTTGTCACCTTCCA | Cloning |
|  | mdr4_rt_for | GGGTGGACAGCGCCAGCGAC | qRT-PCR |
|  | mdr4_rt_rev | GGTCCGGCCTGCCAGGGTCG | qRT-PCR |
|  | sakA_rt_for | CCACGACCCTACCGATGAGCC | qRT-PCR |
|  | sakA_rt_rev | GGCCAGCGTCGTTACCCTGG | qRT-PCR |
|  | crf1_rt_for | CCTACTACCACCGGCGGCAC | qRT-PCR |
|  | crf1_rt_rev | CCGAGCCCTTGATGGAGCCG | qRT-PCR |
|  | rodB_rt_for | CCCTGATCGGTGTCGACGACC | qRT-PCR |
|  | rodB_rt_rev | CGAGAGCAACGCAGGCTGGCG | qRT-PCR |
|  | sitT_rt_for | CGCTCTGTGGGCCGAACGGG | qRT-PCR |
|  | sitT_rt_rev | CGGCCCTCTCCAACCCATCC | qRT-PCR |
|  | rlmA_rt_for | GGGTTCGGCAGGTCCGGTCC | qRT-PCR |
|  | rlmA_rt_rev | CGCCGTTCCCTCGATGCTAGG | qRT-PCR |
|  | mpkA_rt_for | CCTCGTCCGGCAGCAGTCCC | qRT-PCR |
|  | mpkA_rt_rev | GCTTGGGCTCCTCCTGCTTCC | qRT-PCR |
|  | rodA_rt_for | CGTCCGCTTCCCCGTTCCTG | qRT-PCR |
|  | rodA_rt_rev | CCACCGATGAGGTTCTTGAGGG | qRT-PCR |
|  | ptc2_rt_for5 | CCAGGAAGGTCGCACAACACG | qRT-PCR |
|  | ptc2_rt_rev5 | CCTCCCCCTCTTGACACTCC | qRT-PCR |

Primers used to create the *A. fumigatus* mutant strains analyzed in this work. In red are reported the primer tails, which recombine with the marker cassette (hph), in yellow the tails recombining with the pYES2 plasmid, in blue the tail recombining with the *xyl*p promoter region. The numbers reported in the column Nr. are referred to the supplementary S1 Fig.
