# Supplementary material for: Network Modeling Reveals Cross Talk of MAP Kinases during Adaptation to Caspofungin Stress in Aspergillus fumigatus
Source: PLoS One. 2015 Sep 10;10(9):e0136932. doi: 10.1371/journal.pone.0136932 (PMC4565559; doi:10.1371/journal.pone.0136932)
Supplement: S4 Table — In the table, systematic names, standard names, description of functions as well as the corresponding GO-categories are listed. The table also indicates whether these genes were previously reported in literature as being part of the response pathway (see reviews from Rispail et al. 2009 and Hamel et al. 2012)[1, 2]. The FDR adjusted p-values for different comparisons are listed, and are referred to the expression patterns after caspofungin (CAS) induction compared to non-induced conditions. Time points after induction are reported in hours (h). (DOC) [file pone.0136932.s010.doc]

**S4 Table. List of genes selected for network inference.**

| **Name** | **Description** | **GO-Categories** | **Reports in literature*** | **FDR adjusted p-value for** | | | | | | | |
| --- | --- | --- | --- | --- | --- | --- | --- | --- | --- | --- | --- |
|  |  |  |  | **wt /**  **wt_CAS_ 0.5h** | **wt /**  **wt_CAS_ 1h** | **wt /**  **wt_CAS_ 4h** | **wt /**  **wt_CAS_ 8h** | **Δ*sakA* /**  **Δ*sakA*_CAS_1h** | **Δ*sakA* /**  **Δ*sakA*_CAS_4h** | **Δ*mpkA* /**  **Δ*mpkA*_CAS_1h** | **Δ*mpkA* /**  **Δ*mpkA*_CAS_4h** |
| *ags2* | α-1,3-glucan synthase, putative | glucan synthase | no | 1,10E-05 | 8,60E-52 | 2,23E-92 | 6,02E-04 | 5,43E-02 | 5,92E-03 | 4,85E-02 | 1,09E-01 |
| *cla4* | Orthologs have protein serine/threonine kinase activity | protein kinase activity | yes | 7,25E-01 | 5,79E-02 | 1,69E-01 | 4,08E-01 | 6,31E-01 | 8,05E-01 | 5,81E-01 | 3,20E-04 |
| *crf1* | extracellular cell wall glucanase Crf1/allergen | cell wall | no | 2,58E-10 | 1,57E-88 | 4,93E-91 | 4,16E-11 | 9,56E-06 | 3,54E-06 | 1,65E-01 | 2,27E-11 |
| *egnE1* | mutanase | glucan synthase | no | 2,12E-03 | 4,49E-77 | 1,17E-274 | 1,02E-13 | 3,16E-44 | 1,61E-191 | 2,86E-02 | 6,42E-14 |
| *egnE2* | mutanase | glucan synthase | no | 1,02E-06 | 1,29E-89 | 6,86E-115 | 4,69E-05 | 7,39E-01 | 9,10E-01 | 9,53E-01 | 9,51E-01 |
| *exg12* | β-glucosidase, putative | β-glucosidase | no | 1,69E-05 | 1,59E-32 | 9,44E-102 | 7,58E-11 | 3,78E-03 | 9,04E-02 | 2,98E-06 | 4,37E-06 |
| *exg13* | β-D-glucoside glucohydrolase | β-glucosidase | no | 2,89E-05 | 3,67E-38 | 8,46E-106 | 4,98E-18 | 2,51E-17 | 6,03E-12 | 3,65E-01 | 6,49E-08 |
| *exg17* | β-glucosidase, putative | β-glucosidase | no | 7,54E-01 | 2,94E-07 | 5,77E-36 | 2,26E-03 | 4,98E-01 | 4,99E-01 | 3,26E-01 | 2,08E-01 |
| *fks1* | 1,3-β-glucan syntase | 1,3-β-D-glucan synthase | yes | 1,00E+00 | 1,11E-05 | 3,62E-09 | 9,44E-01 | 2,65E-02 | 1,07E-02 | 1,04E-09 | 7,70E-01 |
| *gel3* | 1,3-β-glucanosyltransferase Gel3 | membrane | no | 1,00E+00 | 2,91E-02 | 8,63E-09 | 8,36E-02 | 1,09E-08 | 1,37E-21 | 7,34E-07 | 1,66E-20 |
| *hnm1* | amino acid permease, putative | transmembrane transport | no | 4,52E-08 | 6,02E-12 | 9,71E-05 | 7,96E-02 | 1,15E-06 | 8,38E-02 | 2,71E-03 | 3,34E-01 |
| *mae1* | C4-dicarboxylate transporter/malic acid transport protein, putative | transmembrane transport | no | 2,24E-02 | 4,18E-54 | 1,69E-162 | 2,01E-05 | 1,82E-42 | 1,09E-69 | 9,38E-01 | 9,99E-05 |
| *mdr4* | ABC multidrug transporter | transmembrane transport | no | 2,08E-06 | 1,54E-43 | 1,31E-04 | 3,73E-01 | 1,97E-37 | 3,75E-11 | 2,03E-03 | 5,71E-01 |
| *mirC* | Putative siderophore transporter | ransmembrane transport | no | 3,35E-04 | 1,38E-02 | 5,87E-06 | 3,28E-01 | 3,80E-34 | 1,27E-30 | 2,29E-15 | 2,57E-10 |
| *mpkA* | MAPK; expression increased by cell wall disturbing compounds | protein kinase activity | yes | 5,77E-02 | 7,17E-01 | 8,30E-03 | 2,80E-01 | 6,46E-02 | 4,50E-01 | 3,05E-01 | 9,10E-01 |
| *ptcH* | Predicted catalytic activity, phosphatase | catalytic activity | yes | 9,05E-01 | 1,80E-02 | 1,25E-05 | 7,09E-02 | 2,70E-02 | 5,04E-04 | 1,46E-02 | 5,81E-03 |
| *rck2* | calcium/calmodulin-dependent protein kinase, putative | protein kinase activity | no | 2,63E-16 | 1,45E-05 | 2,53E-28 | 3,87E-14 | 6,31E-01 | 6,17E-01 | 8,26E-02 | 3,12E-13 |
| *rho1* | Putative GTPase; involved in radial growth and conidiation | membrane | yes | 9,05E-01 | 1,28E-06 | 4,51E-04 | 8,67E-01 | 2,17E-01 | 4,99E-01 | 3,65E-01 | 6,41E-01 |
| *rlmA* | Orthologs have role in cell wall organization, cellular response to stress, regulation of transcription | transcription factor | yes | 2,47E-01 | 8,24E-05 | 5,80E-05 | 7,57E-01 | 9,24E-01 | 5,58E-01 | 3,68E-01 | 9,11E-01 |
| *rodA* | conidial hydrophobin | cell wall | no | 5,49E-02 | 6,64E-01 | 6,82E-38 | 8,25E-04 | 7,77E-01 | 1,00E+00 | 4,07E-01 | 9,63E-02 |
| *rodB* | conidial hydrophobin | cell wall | no | 1,66E-05 | 3,85E-58 | 8,02E-250 | 3,99E-11 | 2,44E-144 | 1,10E-131 | 4,31E-01 | 5,95E-01 |
| *sakA* | MAP kinase | protein kinase activity | yes | 7,84E-03 | 4,91E-05 | 2,81E-10 | 1,72E-03 | Na | Na | 1,22E-02 | 3,24E-10 |
| *sitT* | ABC multidrug transporter | transmembrane transport | no | 2,66E-10 | 2,68E-79 | 3,18E-53 | 2,07E-02 | 9,83E-113 | 2,72E-118 | 3,52E-27 | 6,09E-28 |
| *ssk1* | Putative response regulator, part of a two-component signal transduction system | transcription factor for ssk2 | yes | 6,92E-01 | 1,00E+00 | 1,57E-03 | 4,54E-01 | 5,85E-12 | 2,41E-08 | 4,21E-09 | 1,97E-12 |
| *ssk2* | Orthologs have role cellular response to osmotic stress | protein kinase activity | yes | 3,04E-03 | 2,67E-04 | 5,50E-01 | 2,17E-01 | 7,06E-02 | 5,38E-01 | 7,55E-01 | 3,16E-01 |
| *ypd1* | Putative histidine-containing phosphotransfer intermediate protein | signal transduction | yes | 1,25E-01 | 7,38E-03 | 1,15E-02 | 3,44E-02 | 9,49E-01 | 4,92E-01 | 8,93E-01 | 5,64E-01 |

In the table, systematic names, standard names, description of functions as well as the corresponding GO-categories are listed. The table also indicates whether these genes were previously reported in literature as being part of the response pathway (see reviews from Rispail *et al.* 2009 and Hamel *et al.* 2012). The FDR adjusted p-values for different comparisons are listed, and are referred to the expression patterns after caspofungin (CAS) induction compared to non-induced conditions. Time points after induction are reported in hours (h).

1. Rispail N, Soanes DM, Ant C, Czajkowski R, Grunler A, Huguet R, et al. Comparative genomics of MAP kinase and calcium-calcineurin signalling components in plant and human pathogenic fungi. Fungal Genet Biol. 2009;46(4):287-98.

2. Hamel LP, Nicole MC, Duplessis S, Ellis BE. Mitogen-activated protein kinase signaling in plant-interacting fungi: distinct messages from conserved messengers. The Plant cell. 2012;24(4):1327-51.
