# Supplementary material for: Network Modeling Reveals Cross Talk of MAP Kinases during Adaptation to Caspofungin Stress in Aspergillus fumigatus
Source: PLoS One. 2015 Sep 10;10(9):e0136932. doi: 10.1371/journal.pone.0136932 (PMC4565559; doi:10.1371/journal.pone.0136932)
Supplement: S5 Table — Signals of kinases phosphorylation and the γ-tubulin (the standard loading control) were quantified by the software Bio-1D (Vilber Lourmat Deutschland GmbH) and the MAP kinase/ γ-tubulin ratio was calculated. Data are presented as the means ± S.E. from three independent experiments. In green are highlighted values where the change of the relative signal was > 1.5, while in orange are reported changes < 0.66. In all experiments, the not induced wild type strain CEA10 was used as control reference, with the exception of the western blots performed to evaluate the effects of the silencing of the fks1 gene (shift xylose/glucose); in this case the signal detected in the recipient strain ΔakuB growth on xylose served as reference. (DOC) [file pone.0136932.s011.doc]

| **Relative MpkA phosphorylation** | | | | | | |
| --- | --- | --- | --- | --- | --- | --- |
| **Sample** | **Time (min after induction)** | | | | | |
|  | 0 | 30 | 60 | 120 | 240 | 480 |
| CEA10 CAS 0.1 μg/ml | 1.00 | 0.49 ± 0.12 | 0.89 ± 0.17 | 0.95 ± 0.02 | 0.90 ± 0.04 | 1.52 ± 0.15 |
| CEA10 CAS 1 μg/ml | 1.00 | 0.67 ± 0.01 | 0.84 ± 0.08 | 1.08 ± 0.11 | 0.99 ± 0.03 | 2.05 ± 0.08 |
| CEA10 CAS 10 μg/ml | 1.00 | 1.29 ± 0.14 | 0.68 ± 0.05 | 1.15 ± 0.13 | 1.70 ± 0.42 | 0.96 ± 0.05 |
| CEA10 CAS 20 μg/ml | 1.00 | 0.63 ± 0.05 | 1.09 ± 0.04 | 2.83 ± 0.70 | 4.15 ± 0.39 | 4.42 ± 1.25 |
| Δ*akuB* 0.1 μg/ml | 0.74 ± 0.09 | 0.46 ± 0.06 | 1.23 ± 0.07 | 1.23 ± 0.05 | 1.53 ± 0.05 | n.d. |
| Δ*ptcH* 0.1 μg/ml | 0.57 ± 0.05 | 0.31 ± 0.02 | 0.29 ± 0.04 | 0.25 ± 0.04 | 0.56 ± 0.04 | n.d. |
| Δ*sakA* 0.1 μg/ml | 0.48 ± 0.04 | 0.19 ± 0.01 | 0.24 ± 0.02 | 0.62 ± 0.03 | 0.51 ± 0.04 | n.d. |
| Δ*akuB* shift xylose/glucose | 1.00 | 0.94 ± 0.05 | 0.99 ± 0.03 | 0.88 ± 0.03 | 1.29 ± 0.05 | 1.33 ± 0.03 |
| *xyl*p-*fks1* shift xylose/glucose | 1.00 | 1.18 ± 0.01 | 1.33 ± 0.03 | 1.42 ± 0.05 | 1.65 ± 0.03 | 1.83 ± 0.05 |
| **Relative SakA phosphorylation** | | | | | | |
| **Sample** | **Time (min after induction)** | | | | | |
|  | 0 | 30 | 60 | 120 | 240 | 480 |
| CEA10 CAS 0.1 μg/ml | 1.00 | 0.44 ± 0.03 | 0.45 ± 0.06 | 0.80 ± 0.04 | 0.83 ± 0.05 | 0.72 ± 0.02 |
| CEA10 CAS 1 μg/ml | 1.00 | 0.91 ± 0.02 | 0.56 ± 0.03 | 1.60 ± 0.16 | 0.36 ± 0.20 | 2.83 ± 0.40 |
| CEA10 CAS 10 μg/ml | 1.00 | 2.14 ± 0.20 | 0.36 ± 0.10 | 0.91 ± 0.03 | 0.93 ± 0.05 | 0.21 ± 0.03 |
| CEA10 CAS 20 μg/ml | 1.00 | 0.78 ± 0.02 | 1.42 ± 0.05 | 2.91 ± 0.08 | 3.76 ± 0.24 | 1.41 ± 0.26 |
| Δ*akuB* 0.1 μg/ml | 0.51 ± 0.05 | 0.31 ± 0.02 | 0.29 ± 0.04 | 0.25 ± 0.04 | 0.56 ± 0.04 | n.d. |
| Δ*ptcH* 0.1 μg/ml | 0.35 ± 0.02 | 0.22 ± 0.01 | 0.15 ± 0.01 | 0.21 ± 0.02 | 0.58 ± 0.01 | n.d. |
| Δ*mpkA* 0.1 μg/ml | 3.19 ± 0.16 | 2.71 ± 0.10 | 2.13 ± 0.07 | 2.24 ± 0.04 | 0.74 ± 0.02 | n.d. |
| Δ*akuB* shift xylose/glucose | 1.00 | 0.60 ± 0.04 | 0.58 ± 0.02 | 0.51 ± 0.02 | 5.39 ± 0.26 | 6.27 ± 0.43 |
| *xyl*p-*fks1* shift xylose/glucose | 1.00 | 0.77 ± 0.03 | 0.76 ± 0.05 | 0.76 ± 0.07 | 1.14 ± 0.10 | 0.42 ± 0.10 |

**S5 Table. Statistical analysis of signal intensities obtained during immune blots experiments.**

Signals of kinases phosphorylation and the γ-tubulin (the standard loading control) were quantified by the software Bio-1D (Vilber Lourmat Deutschland GmbH) and the MAP kinase/ γ -tubulin ratio was calculated. Data are presented as the means ± S.E. from three independent experiments. In green are highlighted values where the change of the relative signal was > 1.5, while in orange are reported changes < 0.66. In all experiments, the not induced wild type strain CEA10 was used as control reference, with the exception of the western blots performed to evaluate the effects of the silencing of the *fks1* gene (shift xylose/glucose); in this case the signal detected in the recipient strain Δ*akuB* growth on xylose served as reference.
