# Supplementary material for: Network Modeling Reveals Cross Talk of MAP Kinases during Adaptation to Caspofungin Stress in Aspergillus fumigatus
Source: PLoS One. 2015 Sep 10;10(9):e0136932. doi: 10.1371/journal.pone.0136932 (PMC4565559; doi:10.1371/journal.pone.0136932)
Supplement: S1 Text — (additional details about the used protocol for immunoblot analysis and image acquisition). (DOC) [file pone.0136932.s012.doc]

**S1 Text**

**Mapping of transcription data**

Total reads were provided as FASTA-ﬁles. TopHat v1.4.1 was used with a butterﬂy search to map all reads to the *Aspergillus fumigatus* A1163 sequenced genome provided by the CADRE database. A CADRE gene transfer format (GTF) ﬁle of the same strain was used to annotate the mapped reads. The number of allowed multi-hits was set to 1. After investigation of the GTF ﬁle, the maximum intron length was set to 2,700 bp. The successfully mapped reads were sorted and indexed using SAMtools . The hits were read into R using the easyRNASeq package . The chromosome size was calculated from the CADRE genome sequence ﬁle and the read counts were calculated. BioMart provided exon annotation. The DESeq package was used to normalize the read counts for library size and to perform the differential expression analysis *via* the nbinomTest function. Genes were considered differentially expressed, with an FDR-adjusted p-value ≤0.05. For the wild-type strain, the time points 0.5 h, 1 h, 4 h, 8 h were compared to the control (0 h). The same procedure was used to investigate the differential expressed genes in the Δ*akuB*, Δ*mpkA* and Δ*sakA* mutant strains.

**Network inference**

The Net*Gene*rator version 2.1 was used to infer the model. The normalized, logarithmic read counts were scaled to fall between [−1, +1]. According to this, the expression value of the time point 0 h (control) was counted as 0 and in order to obtain relative expression changes. All three different biological replicas collected for each time point were considered as independent experiments. The implementation of the prior knowledge was obtained by creating two matrices, one for the direction (activating/repressing) and one for the conﬁdence of the explicit interaction (see supplementary Tab. S3 for details).

Three different parameters were adjusted:

*allowedError*: The allowed model error compared to the expression data.

*weightingStruct*: The global weight of the prior knowledge.

*maxDynamicOrder*: The dynamic model order.

The perimeters were checked for the following values:

*allowedError*: 0.001, 0.010, 0.050, 0.100, 0.200, 0.300, 0.400, 0.500, 0.600, 0.700, 0.800, 0.900, 1.000

*weightingStruct*: 0.000, 0.001, 0.010, 0.050, 0.100, 0.200, 0.300, 0.400, 0.500, 0.600, 0.700, 0.800, 0.900, 1.000

*maxDynamicOrder*: 1, 2

The model was investigated according to the number of interactions, number of implemented prior knowledge and model ﬁt.

The susceptibility of the network to the perturbations of data was measured by distributing artificial noise to the pre-processed data (mean = 0 and standard deviation of 0.01). The inference analysis was repeated (1000 times for each interaction) and the number of the obtained interactions counted. All these interactions that were predicted at least 501 times during the inference analysis were considered as robust against noise (> 50%).

Another test was performed to check the dependency of the interactions on prior knowledge. The inference analysis was repeated (once again 1000 times for each interaction), but the 10% of the total prior knowledge was randomly omitted during repetitions. Also in this case, all these interactions that were predicted at least 501 times during the inference analysis were considered as robust to perturbation of the prior knowledge (> 50%). At the end, every single interaction that passed both tests was considered as robust, because not depending either on noise or on the missing prior knowledge.

**Protocol for western blot analysis.**

Conidia from *A. fumigatus* were incubated in AMM for 16 h and then stressed using the appropriate caspofungin concentration. Samples were harvested at different time points and immediately frozen in liquid nitrogen. Mycelia were ground under liquid nitrogen to a fine powder using mortar and pestle. For protein extraction, 0.5 ml lysis buffer (10 % (v/v) glycerol, 50 mM Tris-HCl pH 7.5, 1 % (v/v) Triton X-100, 150 mM NaCl, 0.1 % (w/v) SDS, 5 mM EDTA, 50 mM sodium fluoride, 5 mM sodium pyrophosphate, 50 mM beta-glycerophosphate, 5 mM sodium orthovanadate, 1 mM PMSF, and protease inhibitor (Roche Applied Science, Germany)) were added to the ground mycelium. Extracts were centrifuged at 14,000 g for 15 min at 4 °C. The supernatants were collected and the protein concentrations were determined using the Coomassie Plus Protein Assay (Pierce, Germany). 30 µg of proteins for each sample were separated by 12 % (w/v) SDS-PAGE (Invitrogen, Germany) and transferred to polyvinylidene difluoride (PVDF) membranes (Millipore, Germany), using an iBlot® 2 Gel Transfer Device (Invitrogen), according to the manufacturing instructions. The phosphorylation of MpkA was examined using anti- phospho-p38 MAPK (Thr180/Tyr182) antibody while the SakA phosphorylated form was detected using a phospho-p44/42 MAPK (Erk1/2) (Thr202/Tyr204) antibody (Cell Signalling Technology, USA). The γ-tubulin antibody was used as reference (yN-20) (Santa Cruz biotechnology, Inc.). The phospho-p44/42 and the phospho-p38 antibodies were diluted in blocking solution containing TBS 1X, milk powder 5%, and Tween 20 0.01 %. The phospho-p44/42 antibody was diluted to 1/500, while the phospho-p38 γ-tubulin antibodies were diluted to 1/1000. Secondary antibodies were used after 1/2000 dilution. Signal bands were detected using a horseradish peroxidase (HRP)-conjugated second antibody and LumiGLO reagents (Santa Cruz Biotechnology Inc., USA).

After adding the substrate to membranes, signals were detected using the Fusion FX7 system (Vilber Lourmat Deutschland GmbH) avoiding over exposition. The MAP kinase/ γ-tubulin ratio was calculated using the software Bio-1D (Vilber Lourmat Deutschland GmbH), and reported in the supplementary table S5.

## Supplementary References

1. Gilsenan JM, Cooley J, Bowyer P. CADRE: the Central Aspergillus Data REpository 2012. Nucleic Acids Res. 2012;40(Database issue):D660-D6.

2. Li H, Handsaker B, Wysoker A, Fennell T, Ruan J, Homer N, et al. The Sequence Alignment/Map format and SAMtools. Bioinformatics (Oxford, England). 2009;25(16):2078-9.

3. R Development Core Team. R: A Language and Environment for Statistical Computing. Vienna, Austria : the R Foundation for Statistical Computing. ISBN: 3-900051-07-0. Available online at http://www.R-project.org/.

4. Delhomme N, Padioleau I, Furlong EE, Steinmetz LM. easyRNASeq: a bioconductor package for processing RNA-Seq data. Bioinformatics (Oxford, England). 2012;28(19):2532-3.

5. Kasprzyk A. BioMart: driving a paradigm change in biological data management. Database. 2011;bar049.

6. Anders S, Huber W. Differential expression analysis for sequence count data. Genome Biol. 2010;11(10):R106.

7. Guthke R, Moller U, Hoffmann M, Thies F, Topfer S. Dynamic network reconstruction from gene expression data applied to immune response during bacterial infection. Bioinformatics (Oxford, England). 2005;21(8):1626-34.

8. Toepfer S, Guthke R, Driesch D, Woetzel D, Pfaff M. The NetGenerator Algorithm: Reconstruction of Gene Regulatory Networks. In: Tuyls K, Westra R, Saeys Y, Nowé A, editors. Knowledge Discovery and Emergent Complexity in Bioinformatics. Lecture Notes in Computer Science. 4366: Springer Berlin Heidelberg; 2007. p. 119-30.

9. Weber M, Henkel SG, Vlaic S, Guthke R, van Zoelen EJ, Driesch D. Inference of dynamical gene-regulatory networks based on time-resolved multi-stimuli multi-experiment data applying NetGenerator V2.0. BMC Syst Biol. 2013;7:1.
